# Regulation of immune signal integration and memory by inflammation-induced chromosome conformation

**DOI:** 10.1073/pnas.2600693123

**Published:** 2026-09-15

**Authors:** Bence Daniel, Andy Y. Chen, Katalin Sandor, Wenxi Zhang, Zhuang Miao, Kathryn E. Yost, Caleb A. Lareau, Howard Y. Chang, Ansuman T. Satpathy

**Affiliations:** ^a^4D Nucleome Consortium, National Institutes of Health, Bethesda, MD 20892; ^b^ Department of Pathology, Stanford University, Stanford, CA 94305; ^c^ Center for Personal Dynamic Regulomes, Stanford University, Stanford, CA 94305; ^d^ Department of Bioengineering, Stanford University, Stanford, CA 94305; ^e^ HHMI, Stanford University, Stanford, CA 94305; ^f^ Parker Institute for Cancer Immunotherapy, San Francisco, CA 94129

**Keywords:** innate immunity, memory, 3D genome, epigenome

## Abstract

Three-dimensional (3D) genome conformation is central to gene expression regulation, yet our understanding of its contribution to rapid transcriptional responses, signal integration, and memory in immune cells is limited. Here, we study the molecular regulation of the inflammatory response in primary macrophages using integrated transcriptomic, epigenomic, and chromosome conformation data, including base pair-resolution Micro Capture-C. We demonstrate that interleukin-4 (IL-4) primes the inflammatory response in macrophages by stably rewiring 3D genome conformation, juxtaposing endotoxin-, interferon-gamma-, and dexamethasone-responsive enhancers to their cognate gene promoters. CRISPR-based perturbations of enhancer–promoter contacts or CCCTC-binding factor (CTCF) boundary elements show that IL-4-driven conformation changes are required for enhanced and synergistic endotoxin-induced transcriptional responses, as well as transcriptional memory following stimulus removal. Moreover, transcriptional memory mediated by changes in chromosome conformation can occur in the absence of changes in chromatin accessibility or histone modifications. Collectively, these findings demonstrate that rapid and memory transcriptional responses to immunological stimuli are encoded in the 3D genome.

## Significance

Innate immune cells integrate inflammatory cues and retain transcriptional memory, a phenomenon previously attributed mainly to one-dimensional epigenetic mechanisms. We show that inflammatory cytokine exposure remodels the three-dimensional (3D) genome of macrophages by establishing stable chromatin loops that persist after stimulus withdrawal. These “memory loops” connect signal-responsive enhancers with promoters, enabling enhanced and integrated transcriptional responses to subsequent, mechanistically distinct stimuli, even in the absence of canonical epigenetic or transcriptional changes. Using base-pair resolution chromosome conformation mapping and causal CRISPR perturbations, we demonstrate that 3D genome architecture is functionally required for innate immune memory. Our findings uncover a previously unrecognized layer of immune regulation and redefine how inflammatory memory is encoded in chromatin.

Innate immune cells represent the first line of defense against pathogens and evolved to rapidly interpret and respond to changes in their environment. Macrophages (MFs) are one of the most plastic cellular components of the innate immune system and possess the ability to alter their phenotype, for example, by integrating multiple coexisting signals to produce the appropriate inflammatory response, or by establishing immune memory following pathogen clearance (1–3). Traditionally, MF plasticity has been conceptualized along a functional continuum defined by two opposing polarization states: the classically activated M1 state (induced by type 1 inflammatory signals, such as Interferon-gamma—IFNG and/or Lipopolysaccharide—LPS) and the alternatively activated M2 state (induced by type 2 inflammatory signals, such as IL-4/IL-13), although MF states are more heterogeneous under in vivo conditions (4). MF plasticity can be programmed by multiple molecular inputs: 1) cell surface receptors that recognize distinct pathogen-associated molecular patterns, 2) regulation of signal-dependent transcription factors (TFs) downstream of receptor signaling, and/or 3) a highly plastic and permissive epigenetic landscape that encodes transcriptional responses to receptor signaling and TF pathways (2). In the first few hours of inflammation, MFs undergo polarization and change their phenotype by regulating the expression of hundreds of genes (5–9). This rapid transcriptional response is the consequence of a primed epigenetic state, in which accessible, gene-distant regulatory elements (enhancers) are brought near gene promoters to induce gene expression (9–13). Therefore, the three-dimensional (3D) conformation of the genome is presumed to be a critical part of regulating the inflammatory response; however, the relationship between genome architecture and its functional impact on the scale and timing of the inflammatory response, resolution of inflammation, and memory, is understudied.

The genome is organized into large-scale 3D structures, such as compartments and topologically associating domains (TADs—demarcated by the insulator binding TF, CTCF), that separate transcriptionally/epigenetically active (Compartment A) and repressed (Compartment B) genomic regions, and genes with their enhancer elements, respectively (14–16). In MFs, our current understanding supports the notion that compartments and TADs are largely inert to external signals; however, Cohesin and some CTCF boundaries seem to be crucial for signal-driven, rapid transcriptional responses in immune cells, including MFs (17–19). At the level of enhancer–promoter communication, chromatin interactions are considered to be largely preformed (primed) to support quick and robust transcriptional responses to inflammation in innate immune cells (20); however, de novo loop formation has been reported in the context of bacterial infection, inflammation, and interferon-driven MF responses (21–23). These findings indicate that chromatin conformation can exhibit flexibility in a changing microenvironment, such as during an innate immune response, although we lack knowledge—mainly due to technological limitations (i.e., resolution to identify enhancer–promoter loops)—on whether inflammation-induced genome conformation can regulate cellular plasticity and memory formation (24).

Traditional 3C-based approaches, such as Hi-C, can map large-scale architectural features of the genome with high confidence (TADs and compartments), but identification of specific gene-regulatory chromatin loops (i.e., enhancer–promoter contacts) with high-resolution is more challenging (25). Recent studies using micrococcal nuclease-based genome fragmentation following proximity ligation, such as Micro Capture-C (MCC), enabled the generation of base-pair resolution contact maps of specific genomic regions (26, 27). Exploiting these technological advances, we investigated the roles of 3D genome conformation in the setting of IL-4-induced alternative macrophage polarization, inflammatory signal integration, and short-term transcriptional memory formation. IL-4 is a T helper 2 type cytokine that is critical for defense against multicellular parasite infections, drives alternative (M2) MF polarization, and can imprint a stable 1-dimensional (1D) epigenomic program (i.e., histone modifications, TF binding, and chromatin accessibility) during MF polarization with lasting effects to alter the subsequent inflammatory response to endotoxin and interferon (3). Importantly, the interaction between IL-4 and endotoxin can have detrimental, hyperinflammatory effects in the in vivo murine model of airway inflammation and asthma that was suggested to be the result of 1D epigenomic remodeling (3, 8, 9, 12, 28).

In this study, we demonstrate that IL-4 creates >1,000 chromatin loops during MF polarization (in 24-h) and priming, of which, ~40% show short-term memory (stability) after the removal of the cytokine. Of these genome conformational changes, we find hundreds of IL-4-induced chromatin loops that are not associated with 1D epigenomic or transcriptional changes, including those that occur in the proximity of inflammatory genes, such as *Il6*. To study the functional role of IL-4-induced chromatin loops, we establish two treatment paradigms: 1) to study signal integration (two signals are present at the same time) between IL-4 and a secondary, unrelated signal (LPS; IFNG; and Dexamethasone—DEX), and 2) to study short-term transcriptional memory formation that is induced by IL-4 and affects the outcome of MF responses to the above-mentioned secondary signals. We report vastly reprogrammed transcriptional responses following IL-4 treatment that are associated with a remodeled 3D genome conformation of specific gene loci. Using high-resolution MCC analysis to measure promoter interaction profiles of multiple genes, we find that IL-4 primes 3D genome conformation by juxtaposing LPS-, IFNG-, and DEX-activated enhancers to their cognate gene promoters. CRISPR-deletion of the anchor points of IL-4-induced chromatin conformation changes (i.e., enhancers and CTCF-bound insulators) suggests that these de novo genome architectural features are required for enhanced and synergistic LPS-driven transcriptional responses and can provide short-term transcriptional memory. Collectively, our work provides evidence that MF priming, signal integration, and memory can be imprinted in the 3D architecture of the genome.

## Results

### Macrophage Polarization Stably Remodels 3D Genome Conformation.

**Fig. 1.**
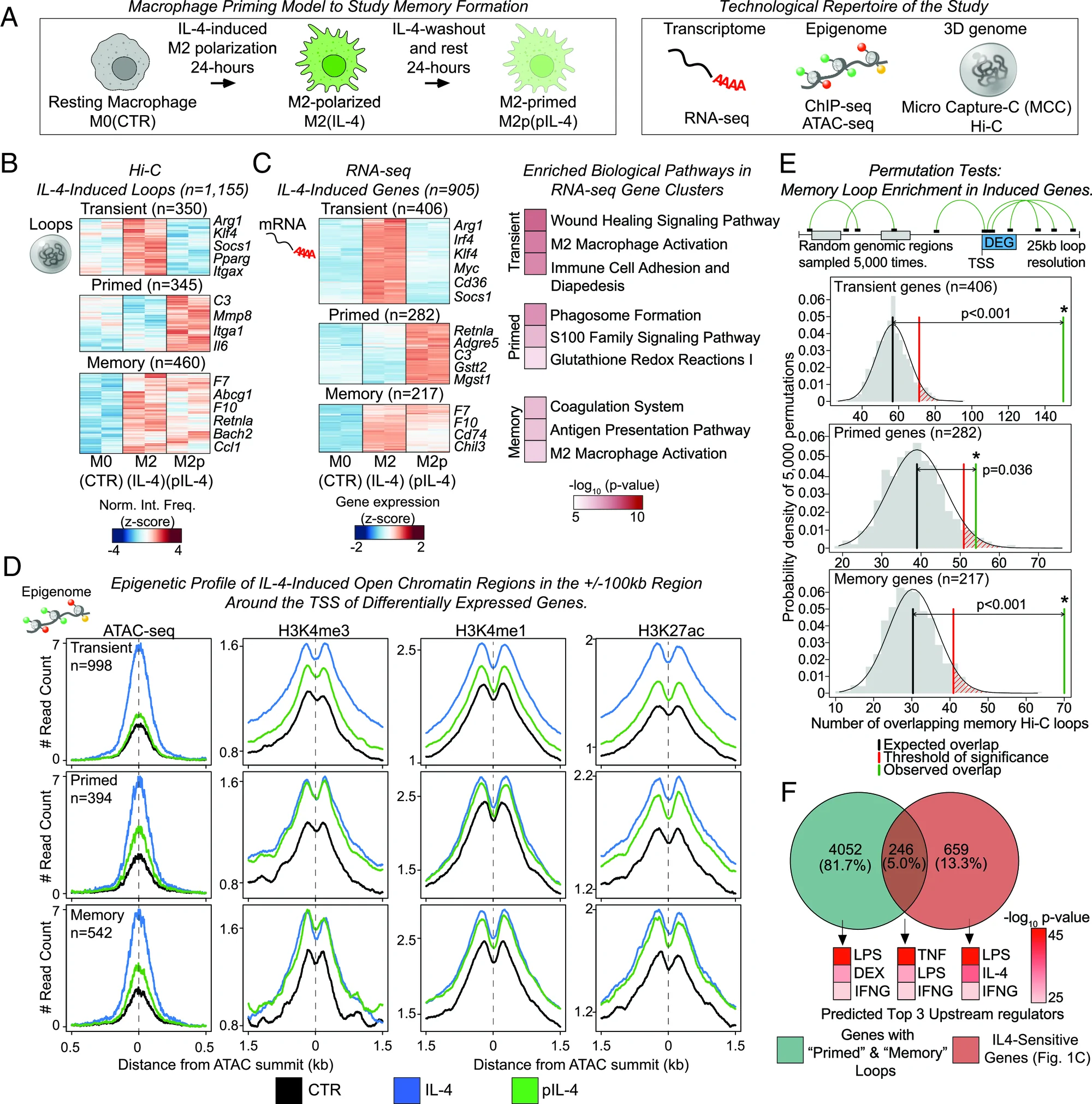
IL-4 remodels chromosome conformation with memory potential. (*A*) Schematic depicting the macrophage priming model used in this study (*Left*). Technological repertoire of the study (*Right*). (*B*) Heatmap representation of differential chromatin loops detected by Hi-C. (*C*) Heatmap representation of DEGs detected by RNA-seq (*Left*). Small heatmaps represent the results of Ingenuity pathway analysis for each RNA-seq gene cluster, and the top 3 biological terms are shown (*Right*). (*D*) Histograms represent the average ATAC-seq and ChIP-seq signal at IL-4 induced open chromatin regions that are in the proximity of DEGs from panel *C*. (*E*) Scheme of the permutation test (*Top*). Permutation test results of the enrichment of memory loops that overlap with DEGs or random genomic regions that are sampled 5,000 times. (*F*) The Venn diagram displays overlap counts between genes associated with Primed/Memory loops and IL-4-sensitive genes and is descriptive rather than an enrichment test. Statistical enrichment of loop anchors near genes in each expression module (Transient, Primed, Memory) is formally assessed by permutation tests against randomly sampled genomic regions matched for genomic distribution (Fig. 1*E* and *SI Appendix*). Ingenuity upstream regulator analysis of the gene sets in the Venn diagram with the top 3 cytokine and chemical drug upstream regulators.

MFs undergo alternative polarization in the presence of IL-4, a cytokine that activates the TF STAT6, and can induce stable, long-term 1D epigenomic changes that impact MF plasticity to unrelated environmental signals via transcriptional memory (8, 9, 28–31). Thus, we hypothesized that IL-4 remodels the 3D genome conformation of MFs, and that this conformational change could also affect MF plasticity. To better understand whether IL-4 impacts genome conformation, we used a MF priming model in which bone marrow–derived MFs (BMDMs) were differentiated for 6 d with M-CSF to a resting M0-like baseline (M0[CTR]). Then, MFs were exposed to IL-4 for 24-h (M2[IL-4]) inducing M2 polarization, followed by cytokine washout and 24-h of rest to obtain primed MFs (M2p[pIL-4]), a model system we extensively characterized previously (32). Using these three conditions, we performed Hi-C, RNA-seq, ATAC-seq, and ChIP-seq for three histone marks that are associated with active chromatin (H3K27ac, H3K4me1, and H3K4me3) to comprehensively profile IL-4-initiated genome conformation, 1D epigenomic, and transcriptomic changes during priming (n = 2 biological replicates; Fig. 1*A*).

Across Hi-C libraries (two biological replicates per condition), we obtained 243.5 to 312.4 million total read pairs per sample, yielding 126.6 to 155.1 million valid unique paired-end reads per sample (average: 141.1 million valid unique pairs). We first performed differential loop calling from Hi-C data (25 kb resolution, Log_2_FC > 0.5, *P*-value < 0.1) and identified a total of 1,818 differential loops among the three conditions, which included 1,155 IL-4-induced and 663 IL-4-repressed loops (Fig. 1*B* and *SI Appendix*, Fig. S1*A*). We next investigated the dynamics of IL-4-induced chromatin loops using k means clustering, which identified three distinct classes of loops based on their behavior across conditions (Fig. 1*B* and *SI Appendix*, Fig. S1*B*). Transient loops (n = 350) were induced during IL-4 stimulation, but interaction frequency returned to baseline after cytokine washout. These loops were associated with canonical M2 genes such as *Arg1, Klf4,* and *Itgax*. In contrast, Primed loops (n = 345) exhibited maximal interaction frequencies following IL-4 withdrawal and were linked to genes including *C3* and *Il6*, consistent with a delayed architectural state. Finally, Memory loops (n = 460) persisted after cytokine removal and were associated with genes such as *F7* and *F10*, indicating stable architectural changes that may contribute to transcriptional memory. We also observed transiently repressed (n = 397) and stably repressed chromatin loops (n = 266; *SI Appendix*, Fig. S1*A*).

Next, we performed differential gene expression analysis from RNA-seq data (FDR < 0.05, Log_2_FC > 0.5) and using k means clustering, identified gene sets with similar expression patterns to the above-described chromatin loop patterns; namely, IL-4-induced Transient genes (n = 406; e.g., *Arg1*, *Klf4*), Primed genes (n = 282; e.g., *C3*, *Mgst1*, *Gstt2*), and Memory genes (n = 217; e.g., *F7*, *F10*, *Chil3*; Fig. 1*C* and Dataset S1). Ingenuity Pathway Analysis showed that Transient genes were enriched for “M2 MF activation”-related terms, Primed genes were enriched for inflammation-associated terms, such as “Phagosome formation” and “S100 Signaling Pathway,” and Memory genes were enriched for coagulation-, antigen presentation-, and M2 MF activation-related terms (Fig. 1*C*). In contrast, IL-4 repressed genes (n = 764) were largely enriched for inflammation-related terms, such as “Toll-like receptor signaling” and “M1 MF activation” (*SI Appendix*, Fig. S1*C* and Dataset S2).

**Fig. 2.**
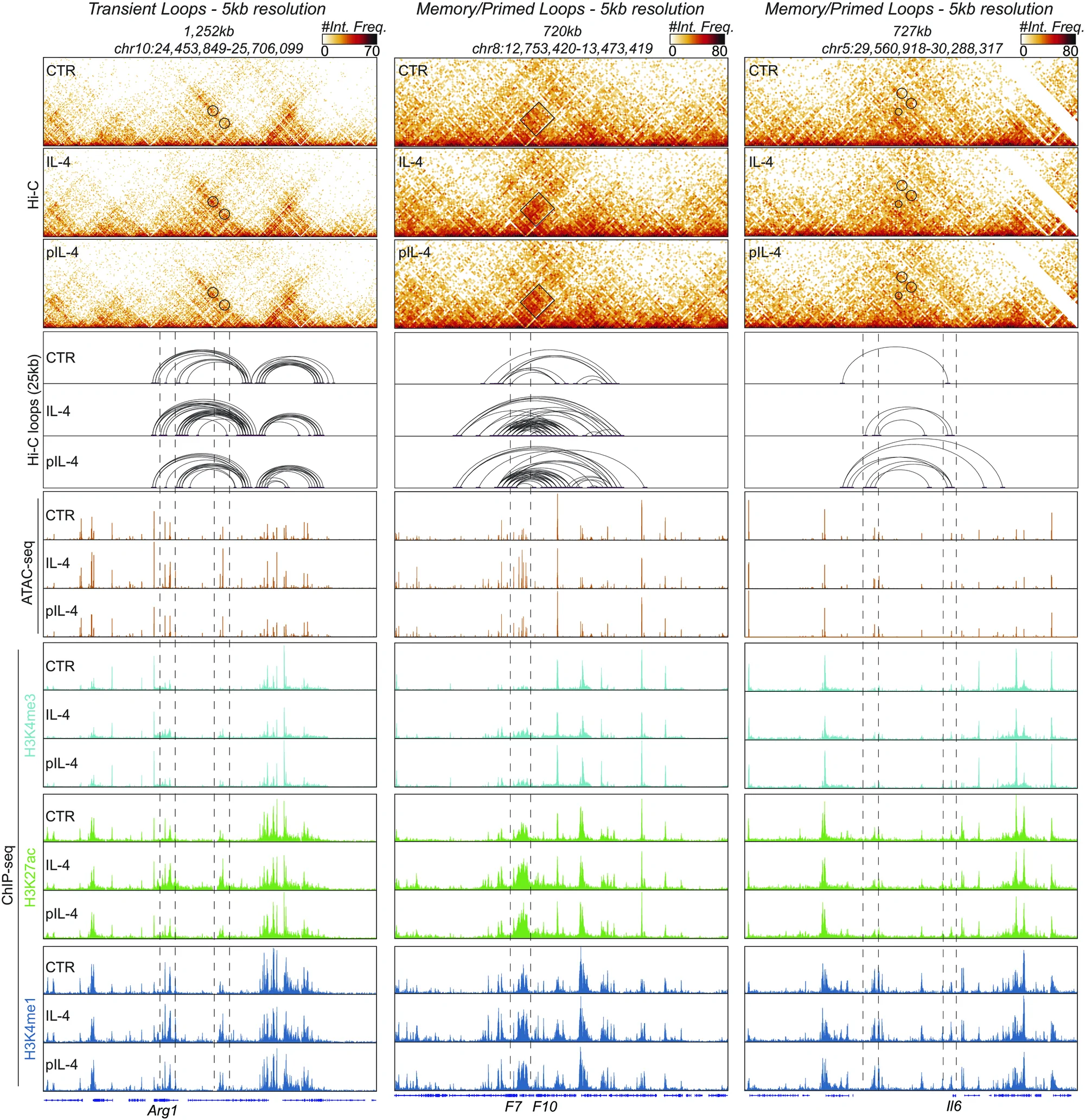
Representative gene loci illustrating Transient, Primed, and Memory chromatin looping states. Hi-C contact maps (5 kb resolution), differential loop calls (25 kb resolution), ATAC-seq, and ChIP-seq profiles (H3K4me1, H3K27ac, and H3K4me3) are shown for representative loci across CTR, IL-4, and pIL-4 conditions. The *Arg1* locus illustrates a Transient looping and epigenetic state that returns to baseline following cytokine washout. The *F7/F10* loci exhibit stable Primed/Memory chromatin loops and persistent histone modification signals after washout. In contrast, the *Il6* locus displays stable IL-4-induced chromatin looping with minimal detectable changes in local chromatin accessibility or histone modifications, consistent with a transcriptionally poised architectural state. Dashed lines indicate representative loop anchor regions. KR (Knight-Ruiz matrix balancing)-normalized contact frequency is shown.

Finally, to characterize the 1D epigenetic changes that occur during IL-4-priming, we performed differential open chromatin region (OCR; *P*-value < 0.05, Log_2_FC > 0.5) and differential histone modification enrichment analyses (H3K27ac, H3K4me3, and H3K4me1; *P*-value < 0.05, Log_2_FC > 0.5) and found similar epigenomic patterns to those observed for gene expression (k means clustering; *SI Appendix*, Fig. S1*D*). To investigate the epigenetic state of the above-described IL-4-induced RNA-seq gene groups, we linked IL-4-induced OCRs (n = 8,269) to these genes (±100 kb around the transcription start sites—TSSs; scheme—*SI Appendix*, Fig. S2*A*), resulting in IL-4-induced OCR sets of Transient (n = 998), Primed (n = 394), and Memory (n = 542) genes. We observed that IL-4-induced chromatin accessibility was largely transient in all three gene groups, and stability, or memory was mainly detected for histone modifications, except for Transient genes, where we observed that all three histone marks exhibited transient patterns (Fig. 1*D*). Of note, among the 1D epigenetic features, H3K4me1 exhibited by far the most stability after washout, retaining ~30% (n = 1,217) of IL-4-induced signal in the genome compared to the other two histone marks (H3K4me3, ~10%, n = 174; H3K27ac, ~19%, n = 794) or open chromatin (~10%, n = 859; *SI Appendix*, Fig. S2*B*). For instance, at the *Arg1* locus (Transient gene), IL-4 induced increases in histone modifications and chromatin accessibility near the TSS and associated enhancer regions, which reverted to baseline following washout. In contrast, at the *F7/F10* loci (Memory genes), IL-4 enhanced histone modification levels that were retained after washout, consistent with a more stable epigenetic state (Fig. 2).

Consistent with these gene-specific patterns, chromatin looping dynamics also differed across loci. At the *Arg1* locus, IL-4–induced loops were transient and reverted after washout, whereas at the *F7/F10* loci, loops persisted, consistent with their classification as Memory loops. Notably, at the *Il6* locus, IL-4 induced chromatin looping that was retained after washout in the absence of detectable changes in chromatin accessibility or histone modifications, indicating that stable 3D genome configurations can form independently of measurable 1D epigenetic remodeling (Fig. 2).

### IL-4-Driven 3D Genome Reprogramming Affects Inflammatory Gene Loci in the Absence of Transcriptional and 1D Epigenomic Changes.

To couple 3D genome conformational changes to gene expression, we performed permutation tests to evaluate whether Memory loops from Hi-C were significantly enriched in the proximity of genes (±100 kb around their transcription start sites) that exhibited Transient, Primed, and Memory gene expression patterns over random genomic regions. Interestingly, we found that Memory loops were enriched in the vicinity of all three gene groups (Fig. 1*E*). IL-4-induced Memory loops were also enriched in the proximity of IL-4-repressed genes (*SI Appendix*, Fig. S2*C*).

Most notably, IL-4-induced Memory loops could be found at IL-4-induced transiently expressed genes. This indicated that despite transient expression, chromatin loops could remain, providing an additional, typically undetected layer of potential gene regulation in response to IL-4 treatment (Fig. 1*E*). To characterize this more broadly, we evaluated the overlap between genes and Memory/Primed loops. Among the 905 IL-4-induced genes, 246 genes overlapped with Memory/Primed loops (e.g., *F7*, *F10*), while among the IL-4-insensitive genes (by expression), 4,052 overlapped with these loops (Fig. 1*F*), including pro-inflammatory genes (e.g., *Il6* with Memory/Primed loops; Figs. 1*B* and 2 and Dataset S3). Next, we examined how many of the 4,052 IL-4-insensitive genes with Primed/Memory loops were associated with IL-4-induced 1D epigenetic changes. We found that 2,463 (~60%) of these genes (±100 kb around the TSSs; OCR—n = 2,046) showed clear 1D epigenetic alterations with IL-4 induced, stable histone marks, suggesting that this gene group undergoes transcriptionally silent 1D and 3D epigenomic reprogramming. In contrast, the remaining ~40% exhibited negligible chromatin accessibility differences (±100 kb around the TSSs; OCR—n = 1,945), and mainly transient histone mark changes, showing that these gene loci only exhibit stability in 3D chromatin reprogramming (*SI Appendix*, Fig. S2*D*).

Last, to predict how IL-4 priming might impact MF response to subsequent stimuli, we performed upstream regulator analysis on the three gene groups defined in Fig. 1*F*. The analysis identified lipopolysaccharide (LPS), dexamethasone (DEX), tumor necrosis factor (TNF), and interferon gamma (IFNG) as the top predicted regulators of gene groups that associate with stable, IL-4-induced chromatin architectural changes. Specifically, IL-4-insensitive genes with Memory/Primed loops contained genes that were predicted to be regulated by LPS, DEX, and IFNG (Fig. 1*F*). Together, these results suggest that chromatin accessibility, histone modifications, and 3D genome reprogramming can be uncoupled from gene expression during MF priming by IL-4, and that IL-4-induced 3D chromatin changes may modulate responses to subsequent environmental signals (e.g., LPS, DEX, IFNG) beyond the already reported effects of 1D epigenetic changes (8, 9, 12, 28).

### IL-4-Driven 3D Genome Conformation is Associated with Enhanced Response to Endotoxin, Interferon Gamma, and Dexamethasone.

**Fig. 3.**
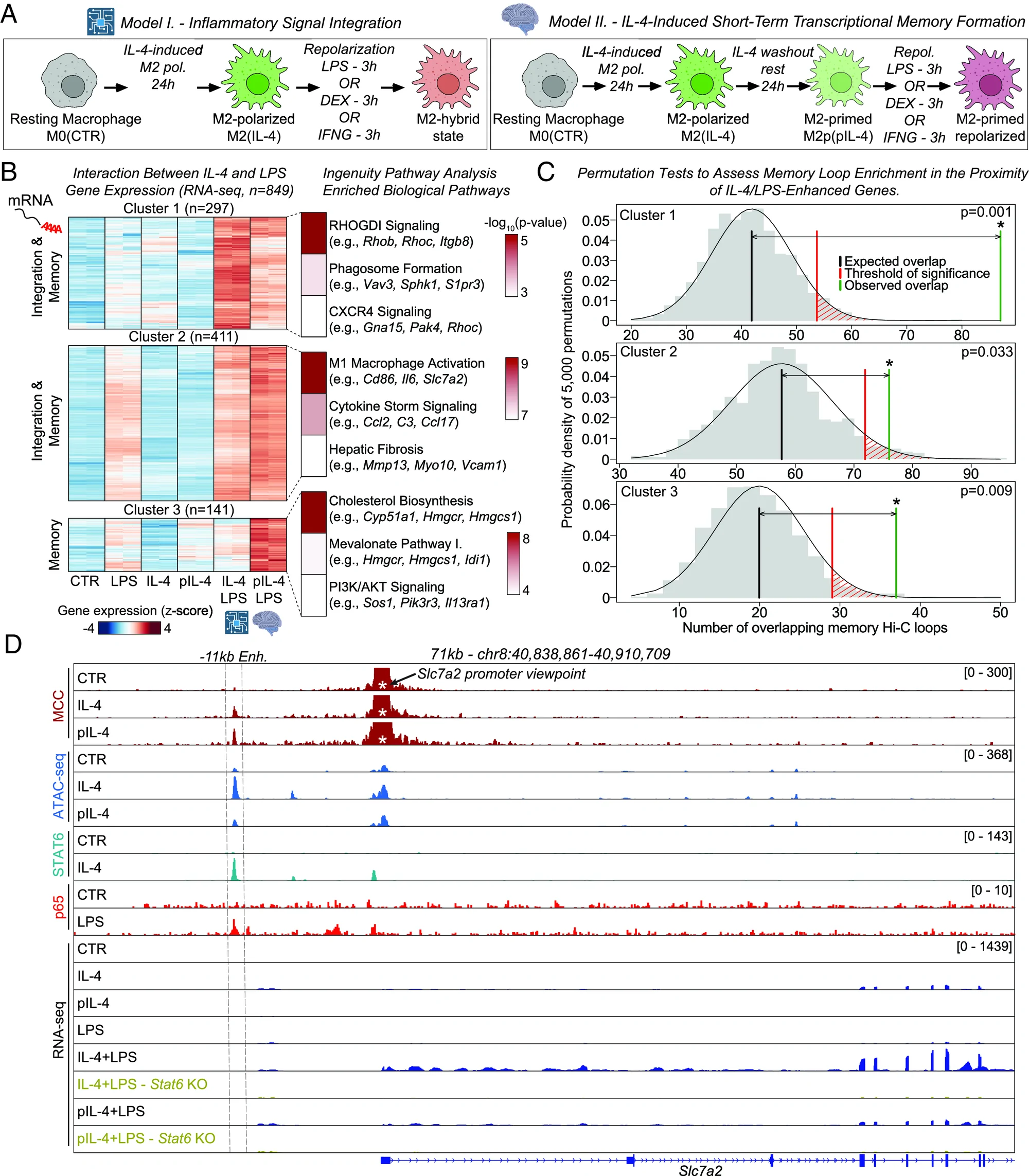
IL-4-induced chromosome conformation juxtaposes signal-responsive enhancers and their cognate promoters. (*A*) Schematic of the two model systems used to study Inflammatory signal integration (Model I.) and Short-term Memory formation (Model II.). (*B*) Heatmap representation of DEGs detected by RNA-seq (*Left*). Small heatmaps represent the results of Ingenuity pathway analysis for each RNA-seq gene cluster, and the top 3 biological terms are shown (*Right*). (*C*) Permutation test results that assess the enrichment of Memory loops in the indicated DEG groups in the IL-4—LPS treatment paradigm. (*D*) Genome browser snapshot of the *Slc7a2* locus. IL-4 priming establishes specific looping interactions between the gene promoter and its cognate enhancer (−11 kb) as detected by MCC. The IL-4-juxtaposed enhancer binds LPS-activated p65 and IL-4-induced STAT6, and this 3D gene regulatory interaction is associated with enhanced transcriptional response to LPS.

To understand the functional impact of IL-4-induced 3D genome conformation on MF responses to environmental challenges, we established two model systems to study: 1) how MFs integrate two inflammatory signals at the same time (Model I.—Inflammatory Signal Integration), and 2) whether IL-4 can imprint short-term transcriptional memory via genome conformation (Model II.—Short-Term Transcriptional Memory; Fig. 3*A* and *SI Appendix*, Fig. S3*A*). We induced M2 MF polarization (24-h IL-4), and then either: 1) immediately treated MFs for 3-h with LPS, IFNG, or DEX (referred to as secondary stimulants) in the presence of IL-4 (Model I.), or 2) washed out IL-4, rested the cells for 24-h and treated with the secondary stimulants (Model II.). We performed bulk RNA-seq in these settings, and we detected hundreds of differentially expressed genes (DEGs) (FDR < 0.05, Log_2_FC > 0.5) with enhanced response across all three secondary stimulants following IL-4 priming in both models. Namely, we observed 849 genes with enhanced LPS response (k means clustering; Cluster 1—strong synergistic response in both models; Cluster 2—enhanced/synergistic response in both models, no IL-4-induced transcription; Cluster 3—strong synergistic response in the model of memory, no IL-4-induced transcription; Fig. 3*B* and Dataset S4), 536 genes with enhanced DEX response (k means clustering; Cluster 1—strong synergistic response in the model of memory, no IL-4-induced transcription; Cluster 2—enhanced/synergistic response in both models; Cluster 3—strong synergistic response in signal integration only; *SI Appendix*, Fig. S3*B* and Dataset S5), and 215 genes with enhanced IFNG response (k means clustering; Cluster 1—enhanced response in priming, no IL-4-induced transcription; Cluster 2—enhanced response in signal integration with partial memory response; *SI Appendix*, Fig. S3*C* and Dataset S6). Genes with an enhanced response after LPS treatment showed enrichment for cell adhesion (Cluster 1—e.g., *Rhob*, *Rhoc*), classical MF activation (Cluster 2—e.g., *Cd86*, *Il6, Slc7a2*), and cholesterol metabolic pathways (Cluster 3—e.g., *Cyp51a1*, *Hmgcr*; Fig. 3*B*). Genes with enhanced DEX response showed enrichment for MF-related inflammatory functions and alternative M2 MF activation (Clusters 2 and 3—e.g., *Klf4*, *Mrc1*, *Fabp4*; *SI Appendix*, Fig. S3*B*). Finally, genes with enhanced IFNG response were enriched for inflammation-related signaling, including cytokine and chemokine pathways (Cluster 2—e.g., *Ccl2*, *Ciita*, *Ccl11*; *SI Appendix*, Fig. S3*C*).

We next performed permutation tests to assess Memory loop enrichment in the proximity of genes exhibiting enhanced response to LPS, DEX, and IFNG. We found that all three gene clusters in the IL-4–LPS (permutation tests: Cluster 1 *P* = 0.001; Cluster 2 *P* = 0.033; Cluster 3 *P* = 0.009; Fig. 3*C*), two in the IL-4–DEX (permutation tests: Cluster 2 *P* = 0.003; Cluster 3 *P* = 0.001; *SI Appendix*, Fig. S3*D*), and an additional group in the IL-4–IFNG (permutation tests: Cluster 2 *P* = 0.001; *SI Appendix*, Fig. S3*E*) treatment settings showed enrichment for Memory loops. In addition to enhanced responses, we identified gene sets where IL-4 treatment and/or priming interfered with secondary stimulant exposure (*SI Appendix*, Fig. S4*A* and Dataset S7). These genes included canonical target genes of our secondary stimulants, such as LPS-induced NF-kB target genes *Tlr2* and *Cd14*, which were both reported as targets of STAT6-mediated direct repression (9). Permutation testing (*SI Appendix*, Fig. S4*B*) revealed that Primed loops were significantly enriched near dampened-response genes (in five of six gene clusters) while Memory loops were not, indicating that IL-4-induced chromatin architectural changes can both enhance and restrict transcriptional responses depending on the class of loop involved. Together, these results indicate that IL-4-induced changes in chromatin conformation are enriched near genes with altered expression upon secondary challenge: Memory loops predominantly associate with enhanced responses, while Primed loops associate with both enhanced and repressed changes in gene expression.

### IL-4-Induced Memory Loops Bridge Signal-Responsive Enhancers and Promoters.

To precisely pinpoint the anchor points of IL-4-induced Memory loops associated with enhanced signal integration or memory response, we performed Micro Capture-C (MCC), a chromosome conformation capture method that can yield base-pair resolution maps of enhancer–promoter interactions (26). We selected loci for high-resolution MCC analysis based on two criteria: 1) significant Memory or Primed loop enrichment by Hi-C, and 2) enhanced transcriptional response to secondary stimuli in the RNA-seq data in a STAT6-dependent manner. These criteria identified gene targets for: 1) enhanced LPS response—*Slc7a2*, *Ccl2*, and *Edn1*; 2) enhanced IFNG response—*Ciita*; and 3) enhanced DEX response—*Klf4,* as candidates for detailed chromatin architecture mapping (Fig. 3*D* and *SI Appendix*, Fig. S5 *A*–*D*). We also performed MCC on two genes whose responses to LPS were dampened by IL-4 (*Cd14* and *Tlr2;* *SI Appendix*, Fig. S6*A*).

MCC revealed remarkably specific IL-4-induced promoter–enhancer contacts that also exhibited stability after cytokine washout for each of these gene promoters. Specifically, we identified IL-4-induced promoter–enhancer contacts in the *Slc7a2* genomic locus with a −11 kb enhancer (Fig. 3*D*), in the *Ccl2* genomic locus with 3 putative enhancers (−28 kb, −12 kb, and +37 kb; *SI Appendix*, Fig. S5*A*), in the *Edn1* locus with a −50 kb enhancer cluster (*SI Appendix*, Fig. S5*B*), and in the *Ciita* genomic locus with a −27 kb enhancer (*SI Appendix*, Fig. S5*D*). Importantly, in the model of short-term transcriptional memory, all these chromatin interactions were either fully retained or further enhanced after IL-4 washout. Closer inspection of these Memory loop anchor points revealed that they closely overlapped with OCRs defined by ATAC-seq, and either showed an IL-4-induced transient change in accessibility (e.g., *Slc7a2*: −11 kb) or did not associate with noticeable changes in chromatin accessibility (e.g., *Klf4*: −92 kb and *Ciita*: −27 kb; *SI Appendix*, Fig. S5). Further, these anchor points showed IL-4-induced STAT6 binding, indicating that STAT6 may be important for the formation of these chromatin loops. Importantly, these same regions readily bound the main effector TFs in the presence of secondary stimulants (i.e., LPS-activated p65 - NF-kB complex; DEX-activated glucocorticoid receptor—GR; and IFNG-activated STAT1). In association with the above-described chromatin conformational changes, we found that the expression profiles of *Slc7a2*, *Ccl2*, *Edn1*, *Klf4*, and *Ciita* (from RNA-seq) showed IL-4-driven induction, which was greatly enhanced by the addition of the secondary stimulants in both models in an IL-4/STAT6-dependent manner (Fig. 3*D* and *SI Appendix*, Fig. S5).

Finally, we examined two loci (*Cd14* and *Tlr2*) that exhibit dampened LPS responsiveness following IL-4 priming (*SI Appendix*, Fig. S6*A*). Similar to loci associated with enhanced responses, MCC identified IL-4-induced promoter-centered chromatin interactions that persisted after cytokine washout and encompassed both Memory and Primed looping configurations. These interactions occurred largely in the absence of strong changes in chromatin accessibility but were associated with STAT6 recruitment following IL-4 stimulation and p65 recruitment following LPS stimulation at multiple regulatory elements. Notably, certain Primed loop anchor regions did not exhibit detectable STAT6 or p65 binding, including the +35 kb element at the *Cd14* locus and the −43 kb element at the *Tlr2* locus, suggesting that additional IL-4-induced TFs may contribute to these interactions.

At the *Tlr2* locus, we also identified IL-4-induced promoter interactions involving STAT6-bound regulatory regions (+29 kb and +19 kb) that did not subsequently recruit p65 upon LPS stimulation. In addition, several promoter-interacting regulatory elements corresponded to previously described STAT6-associated repressive regions identified in IL-4-mediated enhancer repression studies, including the *Tlr2* −21 kb region and the *Cd14* +25 kb and +6.8 kb regulatory elements (*SI Appendix*, Fig. S6*A*) (9). These observations suggest that IL-4-induced chromatin looping may also participate in transcriptionally repressive programs, although the functional contribution of these interactions to gene repression was not directly investigated in the present study.

Together, MCC analysis identified highly specific IL-4-induced promoter-centered chromatin interactions that were stable after cytokine washout and frequently associated with binding of TFs activated by secondary stimuli on a select set of genomic loci. Our results support a model in which IL-4-induced chromatin looping contributes to the integration of distinct inflammatory signals and may have functional relevance in both enhanced and repressive transcriptional responses associated with short-term transcriptional memory.

### IL-4-Driven Chromatin Conformation is Required for Signal Integration and Memory Formation in the *F10* Gene Locus.

**Fig. 4.**
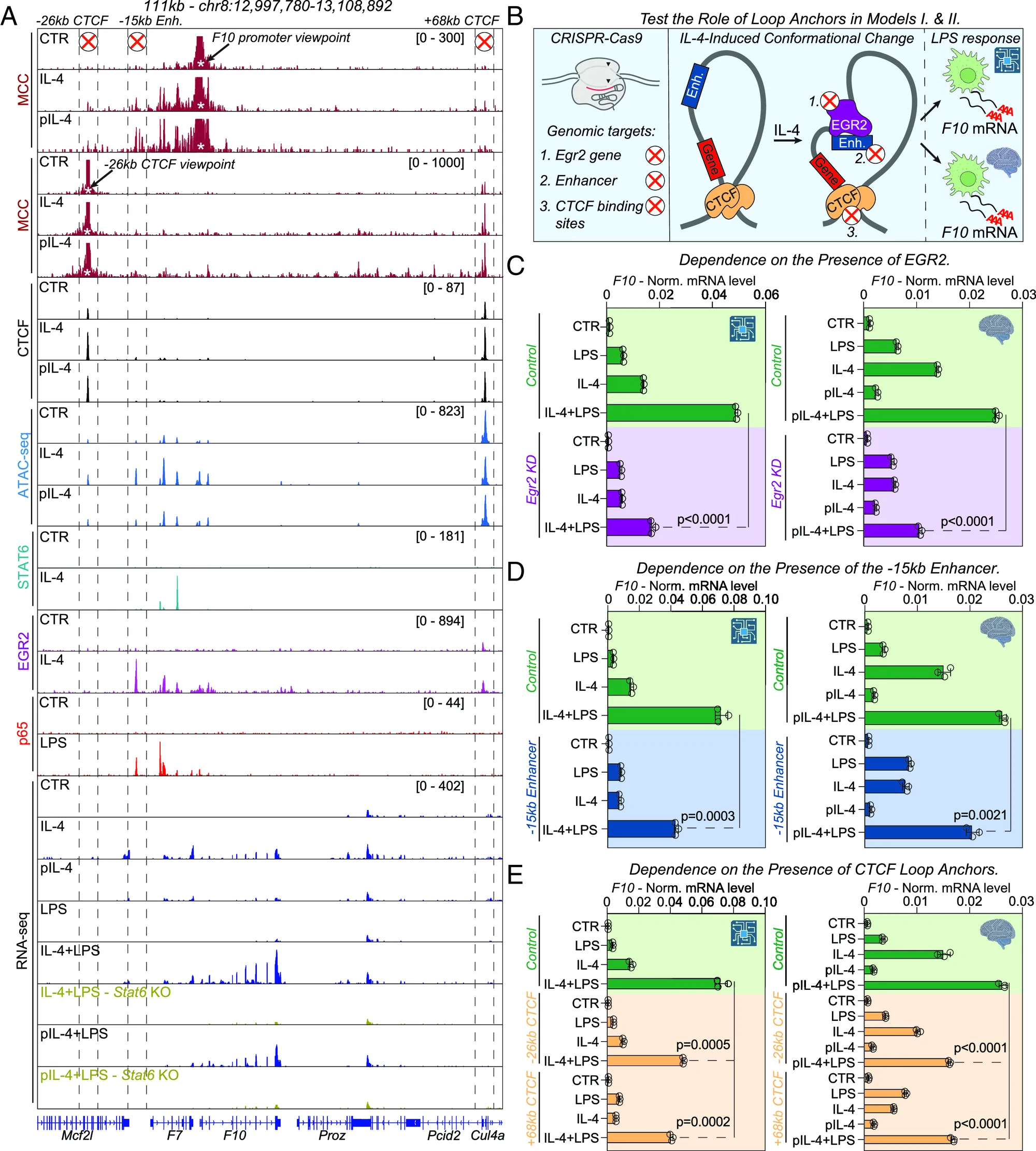
Memory loop anchors are functional, integrate opposing polarization signals, and confer short-term transcriptional memory. (*A*) Genome browser tracks showing the indicated datasets in the *F10* gene locus that nominated three loop anchor points for CRISPR editing. (*B*) Schematic that presents the perturbation strategies used to test the function of IL-4-induced chromatin loop anchors in supporting enhanced LPS response. RT-qPCR measurements of *F10* gene expression in the signal integration (microchip icon) and short-term memory (brain icon) settings upon various CRISPR perturbations: (*C*) *Egr2* knockdown (KD), (*D*) −15 kb enhancer perturbation (same experiment, CTR sample is shared between panels), and (*E*) CTCF loop anchor perturbations (−26 kb and +68 kb). Significant differences were determined by two-tailed, unpaired *t* test at *P* < 0.05, n = 3 biological replicates, and means ± SD is presented. Asterisks (*) indicate the positions of MCC capture oligonucleotides designed to capture interactions from the *F10* promoter region and the −26 kb CTCF-bound regulatory element.

Next, we perturbed IL-4-induced loop anchor points that were associated with inflammatory signal integration and short-term transcriptional memory formation to test their functional role in the IL-4–LPS treatment paradigm in both models. We first focused on *F7* and *F10*, genes located within a locus that exhibited stable IL-4-induced loops in the Hi-C data and are considered as alternative MF polarization markers (Fig. 2). We designed MCC experiments to enrich for *F10* promoter contacts and a CTCF-bound region located −26 kb from the TSS. These experiments recapitulated the Hi-C observations and yielded high-resolution contact maps in the priming system, which revealed IL-4-induced stable looping between the *F10* promoter and not only its cognate enhancers (e.g., −15 kb) but also with two CTCF-bound sites located −26 kb and +68 kb from the TSS. The CTCF-bound sites likely confine this gene regulatory unit and represent the boundaries of a TAD, although with much weaker interaction frequency compared to promoter–enhancer contacts (Fig. 4*A*).

Putative enhancers that were detected in this TAD exhibited strongly elevated ATAC-seq signal in the presence of IL-4 and retained their openness after cytokine washout. Among these enhancers, the −15 kb enhancer readily bound EGR2, an IL-4/STAT6-induced TF with long-term epigenetic effects in IL-4 polarized M2 MFs (8, 28, 33). Additionally, this enhancer also bound LPS-activated p65; however, the most upstream transcriptional regulator of IL-4-induced transcription, STAT6, was not detected at this site, but rather at a neighboring enhancer that is in the intron of the *F7* gene (Fig. 4*A*). This observation aligns with recent reports that STAT6 is an upstream regulator of EGR2’s expression, and that the two TFs’ cistromes (the aggregate of their genome-wide binding sites) are largely mutually exclusive, reflecting spatial and temporal separation during IL-4-driven M2 MF polarization (8, 33). RNA-seq revealed that only *F10* exhibited signal integration and short-term memory formation specifically in the IL-4–LPS setting, while *F7* was insensitive to LPS (Fig. 4*A*). These results nominated the −15 kb enhancer, and the two CTCF sites located −26 kb and +68 kb from *F10* for CRISPR perturbation experiments in primary MFs (Fig. 4*B*).

First, we designed single guide RNAs (sgRNA) to target the *Egr2* gene to assess the requirement of this TF; second, we designed sgRNAs to perturb the −15 kb enhancer; and third, we designed sgRNAs to target the two CTCF sites in both models (Fig. 4*B*). After optimizing electroporation conditions using the cell surface expression of CD11B (ITGAM) as a surrogate readout, according to a recent report [*SI Appendix*, Fig. S6*B*; (34)], we first perturbed *Egr2*, achieving a ~45% reduction in IL-4-induced *Egr2* expression at the mRNA level (two-tailed, unpaired *t* test at *P* < 0.05, n = 3; *SI Appendix*, Fig. S6*C*). This was followed by severely reduced signal integration and short-term transcriptional memory formation in MFs, as measured by RT-qPCR of *F10* mRNA levels (two-tailed, unpaired *t* test at *P* < 0.05, n = 3; Fig. 4*C*). Similarly, we perturbed the −15 kb enhancer, which as a result lost its ability to respond to LPS, as assessed by RT-qPCR measurements of enhancer RNA (eRNA) production (two-tailed, unpaired *t* test at *P* < 0.05, n = 3; *SI Appendix*, Fig. S6*D*). This enhancer was also required, in both treatment paradigms, for the enhanced transcriptional response to LPS, although its perturbation had a milder impact on *F10* gene expression than the loss of *Egr2* (Fig. 4*D*). Finally, we perturbed each of the two CTCF sites (−26 kb and +68 kb), both of which led to reduced signal integration and short-term transcriptional memory formation after IL-4 exposure, as measured by RT-qPCR of *F10* mRNA levels (two-tailed, unpaired *t* test at *P* < 0.05, n = 3; Fig. 4*E*). Collectively, these results indicate that IL-4-induced chromatin conformation, and the anchor points establishing this conformation, are required for enhanced LPS-driven expression of *F10*.

### Transcriptionally Silent, IL-4-Driven 3D Chromatin Conformation Is Required for Enhanced Endotoxin-Induced *Il6* Expression.

Finally, we focused on *Il6*, a pro-inflammatory cytokine and key marker of innate immune memory with central roles in inflammatory responses (24, 35). We observed that *Il6* exhibited enhanced responsiveness to LPS following IL-4 exposure in both signal integration and short-term memory settings. Notably, IL-4 alone did not alter basal *Il6* expression or its local 1D epigenetic state, including chromatin accessibility and histone modifications (Fig. 2), in contrast to loci such as *F10* (Figs. 2 and 4).

**Fig. 5.**
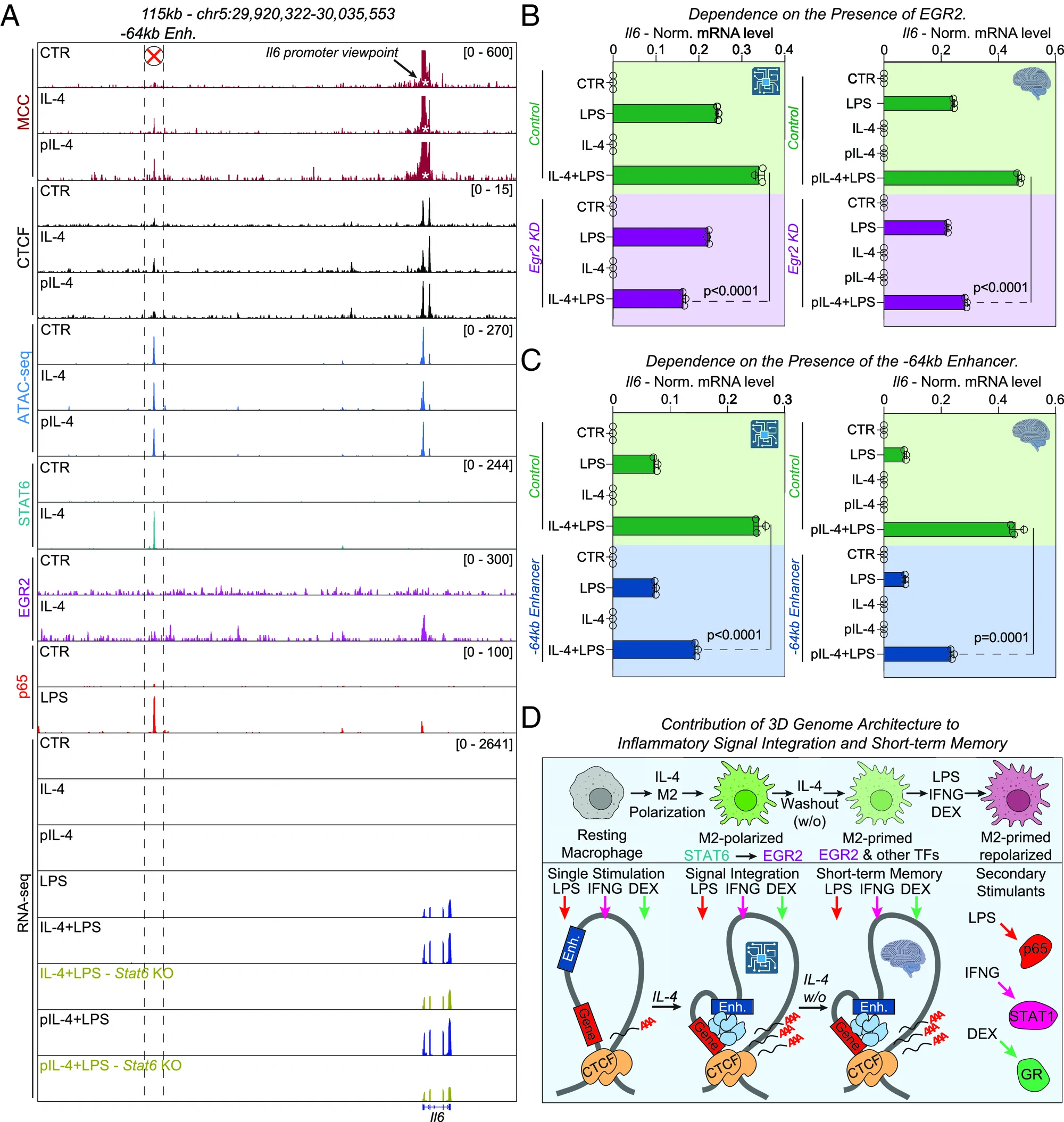
IL-4-induced memory loops can form in the absence of epigenetic remodeling and transcription changes. (*A*) Genome browser tracks showing different datasets in the *Il6* gene locus that nominated a loop anchor point for CRISPR editing. RT-qPCR measurements of *Il6* gene expression in the signal integration (microchip icon) and short-term memory (brain icon) settings upon various CRISPR perturbations: (*B*) *Egr2* knockdown (KD) and (*C*) −64 kb enhancer perturbation. Significant differences were determined by two-tailed, unpaired *t* test at *P* < 0.05, n = 3 biological replicates, and means ± SD is presented. (*D*) IL-4 stimulation induces alternative macrophage polarization and establishes chromatin loops that juxtapose gene promoters with distal regulatory elements. A subset of these interactions persists after cytokine washout in M2-primed macrophages and is associated with enhanced responsiveness to secondary stimulants, including LPS, IFNG, and DEX. Signal-responsive transcription factors, including p65, STAT1, and GR, bind preconfigured regulatory regions during secondary stimulation, contributing to signal integration and altered transcriptional responses. The *Il6* locus exemplifies an epigenetically silent, transcription-independent architectural priming, where 3D chromatin conformation changes might precede and associate with altered transcriptional responses to secondary stimuli.

Using MCC, we identified an IL-4-dependent chromatin loop connecting the *Il6* promoter to a distal putative enhancer element located −64 kb upstream (Fig. 5*A*), consistent with Hi-C observations (Fig. 2). This loop persisted after cytokine washout, indicating stability of the interaction. The distal −64 kb enhancer was accessible and marked by H3K4me1 in resting MFs (Fig. 2), suggesting that loop formation occurs at an epigenetically primed, preexisting regulatory region. At the molecular level, this −64 kb enhancer was bound by STAT6 and EGR2 following IL-4 stimulation and by p65 following LPS stimulation, whereas IL-4 alone promoted the interaction of this element with the *Il6* promoter without detectable changes in chromatin accessibility, histone modifications, or transcription (histone modification—Figs. 2 and 5*A*). These observations indicate that IL-4-induced chromatin looping at the *Il6* locus occurs independently of detectable 1D epigenetic remodeling or gene activation.

To assess the functional relevance of this architectural change, we perturbed *Egr2* and observed a reduction in IL-4-enhanced LPS-induced *Il6* expression in both experimental settings (two-tailed, unpaired *t* test at *P* < 0.05, n = 3; Fig. 5*B*). In addition, CRISPR-mediated targeting of the −64 kb enhancer resulted in decreased eRNA production (two-tailed, unpaired *t* test at *P* < 0.05, n = 3; *SI Appendix*, Fig. S6*E*) and reduced IL-4-dependent enhancement of *Il6* expression following LPS stimulation (two-tailed, unpaired *t* test at *P* < 0.05, n = 3; Fig. 5*C*). At the genome-wide level, 1,414 of 4,298 genes associated with Primed/Memory loops (33%) contained loop anchors that were both ATAC-positive and H3K4me1-positive at baseline, indicating that loop formation frequently occurs at preaccessible and epigenetically primed regulatory elements and suggesting that the *Il6* example may represent a more widespread phenomenon. Together, these results demonstrate that IL-4 can induce stable chromatin interactions at the *Il6* locus without altering basal transcription or local epigenetic state, and that these architectural changes are functionally associated with enhanced responsiveness to subsequent inflammatory stimulation.

## Discussion

Here, we identified a role for 3D genome organization in inflammatory signal integration and short-term transcriptional memory in primary MFs. IL-4 induced widespread remodeling of chromatin architecture, including the formation of hundreds of chromatin loops, a substantial fraction of which persisted following cytokine withdrawal. These Memory loops were enriched near IL-4-regulated genes but also occurred at loci that did not exhibit detectable transcriptional or 1D epigenetic changes, indicating that chromatin architecture can be remodeled independently of local gene activation. Functionally, we showed that genes associated with IL-4-induced chromatin looping exhibited enhanced responsiveness to secondary stimuli, including LPS, DEX, and IFNG. High-resolution MCC analysis revealed that IL-4 induced promoter-centered chromatin interactions that juxtaposed stimulus-responsive enhancer elements with their target genes, and that these configurations persisted after cytokine removal (Fig. 5*D*). Using CRISPR-based perturbations, we demonstrated that these architectural features were required for enhanced transcriptional responses in both signal integration and short-term memory settings. Notably, at the *F10* locus, loop formation was accompanied by transcriptional and epigenetic remodeling, whereas at the *Il6* locus, looping occurred without detectable changes in basal transcription or chromatin state yet remained functionally associated with enhanced responsiveness to LPS. Mechanistically, we identified the STAT6–EGR2 axis as a key regulator of this process. EGR2 is required for IL-4-dependent enhancement of LPS responses, and both EGR2 and LPS-activated p65 bound loop anchor regions, suggesting that these sites function as regulatory hubs for signal integration; however, our perturbation experiments do not allow us to experimentally disentangle enhancer activity from chromatin architecture requirements in this system. Finally, we identified IL-4-induced chromatin interactions involving CTCF-bound elements at the *F10* locus, indicating that both enhancer-associated and architectural loop anchors can contribute to transcriptional memory.

Previous studies have shown that IL-4 or other priming signals can reprogram the MF epigenetic landscape; however, in the absence of high-resolution chromatin conformation mapping, the contribution of chromatin looping to these processes has remained unclear (3, 36, 37). The enhanced LPS-induced expression of *Il6* following β-glucan (BG) priming is a hallmark of trained innate immunity, and our findings suggest that a similar 3D genome priming mechanism may operate in this context, although IL-4 and BG differ in their effects on basal gene expression. Consistent with this, IL-4 priming promoted LPS-driven activation of cholesterol metabolism and the mevalonate pathway, key metabolic programs implicated in trained immunity (38). We further observed that IL-4-activated STAT6 induced EGR2 and MITF expression (8, 33), paralleling transcriptional programs observed in β-glucan-trained MFs (37). In our system, the STAT6–EGR2 axis was required for enhanced LPS responsiveness, suggesting that this transcriptional pathway contributes to the formation or function of memory-associated chromatin loops and may represent a shared regulatory feature of innate immune memory.

Recent studies suggest that active transcription and enhancer activation can, in some contexts, drive 3D chromatin conformational changes, although the temporal and mechanistic relationships between these processes remain unresolved (39–42). Prior studies have shown that signal-dependent TFs can promote chromatin looping at regulatory elements, including STAT5-driven interactions within super enhancer regions in T cells (43) and multi-enhancer interaction hubs during MF differentiation (22). In contrast to these systems, we found that IL-4 could induce chromatin looping at the *Il6* locus without detectable changes in local chromatin accessibility, histone modification, or basal transcription. These observations suggest that, in some contexts, 3D genome reorganization may precede or occur independently of canonical enhancer activation and instead establish a permissive architectural configuration that facilitates enhanced responsiveness to subsequent stimuli. While our data support a functional role for these structures in signal integration and short-term memory, additional regulatory mechanisms likely contribute to these effects.

An important aspect of this study is the use of IL-4-mediated MF priming as a model system. IL-4 induces a well-defined and extensively characterized epigenomic program mediated by the STAT6–EGR2 transcriptional cascade, which can persist after cytokine withdrawal and has been linked to altered transcriptional responsiveness (8, 28, 33). Importantly, prior studies have shown that IL-4 signaling can both repress inflammatory enhancers and reprogram MF responses at the 1D epigenomic level, providing a mechanistic foundation for exploring whether similar regulatory principles operate in 3D genome architecture (9, 28, 32). Together, these features make IL-4 priming a tractable system for investigating how signal-dependent epigenomic remodeling may extend into higher-order genome organization.

Overall, our results indicate that 3D genome conformation in MFs is dynamically regulated during polarization and is associated with the integration of inflammatory signals and the establishment of short-term transcriptional memory. A limitation of this study is that we focused exclusively on IL-4 priming rather than reciprocal IFNG or other priming paradigms on a rather small set of gene loci. In addition, while our analyses primarily focused on IL-4-dependent enhancement of inflammatory responses, IL-4 can also suppress LPS-induced gene expression at specific loci, such as *Cd14* and *Tlr2* (9). Notably, these loci were also associated with primed or memory-like chromatin looping configurations in our dataset, indicating that similar architectural states may provide a shared framework for both activating and repressive regulatory programs. However, because our study was designed to investigate signal integration and enhanced transcriptional responsiveness, the functional contribution of these interactions to gene repression was not systematically explored. Accordingly, we focused on IL-4 priming as a defined model for probing higher-order genome organization in macrophages. Extending these analyses to additional inflammatory contexts will be important for determining the generality of 3D genome–mediated signal integration and memory.

More broadly, our findings suggest that one component of cellular memory may be encoded in specific 3D genome configurations. Computational models have proposed that higher-order chromatin folding can contribute to the maintenance of cell identity through cell division (44), whereas recent live-cell imaging studies indicate that chromatin loops can be highly dynamic and short-lived (45). Together, these observations highlight the need to better understand how transient and stable chromatin interactions contribute to functional regulatory states. Finally, our study focused on short-term transcriptional memory, and an important open question is whether IL-4-induced architectural changes persist over longer time scales (days to weeks) and whether they operate in vivo during physiological inflammatory responses. Given that trained innate immunity paradigms involve durable epigenetic changes lasting weeks to months, determining whether 3D genome conformational memory contributes to such long-term phenomena will require longitudinal studies and in vivo models of macrophage priming.

## Methods Summary

BMDMs were differentiated from mouse bone marrow and subjected to IL-4 stimulation, cytokine washout, and secondary stimulation as described in *SI Appendix*. RNA-seq, ATAC-seq, ChIP-seq, Hi-C, and Micro Capture-C (MCC) were performed using established protocols. Differential gene expression, chromatin accessibility, and chromatin interaction analyses were performed using standard computational pipelines, including DESeq2, HICCUPS, and permutation-based enrichment testing. CRISPR-Cas9 perturbations were performed in primary macrophages to assess the functional contribution of selected loop anchor regions. Primer and guide RNA sequences are reported in Dataset S8. Detailed experimental procedures, sequencing analyses, and statistical methods are provided in *SI Appendix*.

## Supplementary Material

Appendix 01 (PDF)

Dataset S01 (XLSX)

Dataset S02 (XLSX)

Dataset S03 (XLSX)

Dataset S04 (XLSX)

Dataset S05 (XLSX)

Dataset S06 (XLSX)

Dataset S07 (XLSX)

Dataset S08 (XLSX)

## Data Availability

Sequencing data are deposited to GEO under accession: GSE247946 (46). All other data are included in the manuscript and/or supporting information.
