# Supplementary material for: Regulation of immune signal integration and memory by inflammation-induced chromosome conformation
Source: Proc Natl Acad Sci U S A. 2026 Sep 15;123(38):e2600693123. doi: 10.1073/pnas.2600693123 (PMC13598234; doi:10.1073/pnas.2600693123)
Supplement: Supplementary file 1 [file pnas.2600693123.sapp.pdf]

Supporting Information for

Regulation of immune signal integration and memory by inflammation-induced chromosome conformation.

Bence Daniel[1,2,3,7,8,#], Andy Y. Chen[1,2,6,7], Katalin Sandor[1,2,7,12], Wenxi Zhang[1,2], Zhuang Miao[1,2], Kathryn E. Yost[3,10], Caleb A. Lareau[1,2,9], Howard Y. Chang[3,4,11] and Ansuman T. Satpathy[1,2,5,#]

Bence Daniel and Ansuman T. Satpathy
Email: daniel.bence@gene.com or satpathy@stanford.edu

This PDF file includes:

Supporting text for Methods
Figures S1 to S6
Legends for Datasets S1 to S8
SI References

Other supporting materials for this manuscript include the following:

Datasets S1 to S8

Methods

Mice and macrophage differentiation

Wild type, 2-3 months old female C57BL/6 mice were purchased from Jackson Laboratories. Female *Stat6*$^{-/-}$ mice were purchased from Jackson Laboratories. Mice were sacrificed, and bone marrow was isolated from the tibiae and femora of the animals. Red blood cell lysis was carried out, and cells were plated in differentiation media containing 10% FBS, Dulbecco's Modified Eagle's Medium (DMEM), and 20ng/ml mouse M-CSF (Peprotech). On the third day of differentiation, media was replaced with fresh differentiation media. Cytokine treatments and sorting procedures were carried out on the 6th day of differentiation.

Treatment conditions

On the 6th day of bone marrow-derived macrophage differentiation, cells were either left untreated or were exposed to mouse IL-4 (20ng/ml; Peprotech) for 24-hours. For testing signal integration and short-term memory formation, following the 24-hour IL-4 treatment, cells were either left in IL-4 and immediately treated for 3-hours with the secondary stimulants (LPS – 100ng/ml, DEX – 100nM, IFNG – 20ng/ml), or IL-4 washout was performed (3-times with PBS after the removal of IL-4 containing media), and cells were further incubated for 24-hours in differentiation media followed by secondary stimulant treatment for 3-hours, respectively.

CRISPR-based gene and *cis*-element engineering

On the 5th day of bone marrow-derived macrophage differentiation, cells were scraped in differentiation media, spun, and resuspended and counted in PBS. Before the cells were prepared and counted for electroporation, Cas9-Ribonucleoprotein (RNP) complexes were prepared as follows: crRNAs and tracrRNA were resuspended to 100uM in resuspension buffer (IDT). For each electroporation reaction, 2ul crRNA was mixed with 2ul tracrRNA for duplex formation. Duplex formation was carried out as follows: crRNA/tracrRNA complexes were heated to 95C for 5-minutes followed by cooling to 20C at 0.5C per second and were stored on ice until it was mixed with recombinant Cas9 at 3:1 molar ratio. RNP complexes were made in the following reaction while incubating at room temperature for 20-minutes: 4ul RNA duplex, 1.33ul 10mg/ml recombinant Cas9, and 1ul H_2O. After the Cas9-RNP complexes were made, macrophages were scraped and counted to achieve 0.5x10^6 cells and prepared in 20ul P3 buffer for electroporation (LONZA - 4D Nucleofector). To each 20ul macrophage suspension in P3 buffer, 5ul of Cas9-RNP complex solution was added and mixed 5-times before 24ul was transferred to an electroporator well (pipetted two times in 12ul volumes). Electroporation was performed with the CM-137 setting as described previously (1). Immediately after electroporation, 180ul differentiation media (including 20ng/ml M-CSF) that was pre-warmed to 37C was added to each well, and cells were resuspended and plated to 12 well plates in a total of 400ul macrophage differentiation media. Treatments were performed on the next day as described above in the Treatment conditions section that was followed by

incubation and either flow cytometry or RNA isolation and real-time quantitative PCR measurements. Guide RNA sequences are available from Dataset S8.

RNA isolation
RNA was isolated from $2x10^5$ bone marrow-derived macrophages (BMDMs) by standard, TRIzol-based RNA precipitation method as follows. Cells were resuspended in 1ml TRIzol (Ambion). Chloroform was added (200ul) to this lysate and extensively vortexed to achieve a homogenous mixture; then, it was incubated for 3-minutes at room temperature before centrifugation at 14,000 rcf (relative centrifugal force) at 4C for 15-minutes. The aqueous layer was collected from the top and transferred into a new tube (~550ul), 1ul GlycoBlue (Ambion) was added, and the RNA was precipitated with equal volume of 2-propanol for 20-minutes at room temperature. RNA precipitates were centrifuged at 16,000 rcf for 15-minutes at 4C and supernatant was carefully discarded without disturbing the GlycoBlue-stained, blue, RNA pellet. The RNA pellet was washed with 1ml 75% EtOH and after the wash, dissolved in 30ul nuclease-free water. RNA concentration was determined by nanodrop, and RNA quality was determined by Agilent Bioanalyzer.

Real-Time quantitative PCR (RT-qPCR)
RNA was isolated with Trizol reagent (Ambion). RNA was reverse transcribed with High-Capacity cDNA Reverse Transcription Kit (Applied Biosystems) according to the manufacturer's instructions. Transcript quantification was performed by qPCR reactions using SYBR green master mix (BioRad). Transcript levels were normalized to *Ppia*. Primer sequences are available from Dataset S8.

Flow cytometry
Macrophages (~0.5×10^6) were treated with Fc-block (1:100) for 15-minutes on ice and then stained with anti-ITGAM (rat monoclonal BV-510-conjugated, BioLegend) in a 1:200 dilution in FACS buffer (0.1% BSA, 2mM EDTA, 5% FBS in PBS) for 20-minutes on ice. Cells were spun and resuspended in PBS with LIVE/DEAD Fixable Aqua cell stain and incubated for 10-minutes on ice followed by FACS analysis.

RNA-seq
Approximately 50ng RNA was reverse-transcribed to complementary DNA (cDNA) and second strand was synthesized by the Ovation RNA-seq System V2 (Tecan) according to the manufacturer’s recommendations. Double-stranded DNA was subjected to isothermal amplification and was purified with Ampure XP beads. DNA was quantified by Qubit, and 80 ng DNA was used for sequencing library construction with the Ovation Ultralow Library System V2 (Tecan) using 8 PCR cycles according to the manufacturer’s recommendations. Libraries were sequenced with Illumina Novaseq 6000 using a paired-end 75bp read configuration.

ChIP-seq

ChIP-seq was performed as previously described with the following modifications (2). Bone marrow-derived macrophages (5 x 10^6) were double crosslinked by 50mM DSG (disuccinimidyl glutarate, #C1104 - ProteoChem) for 30-minutes followed by 10-minutes of 1% formaldehyde. Formaldehyde was quenched by the addition of glycine. Nuclei were isolated with ChIP lysis buffer (1% Triton x-100, 0.1% SDS, 150 mM NaCl, 1mM EDTA, and 20 mM Tris, pH 8.0). Nuclei were sheared with a Covaris sonicator using the following setup: Fill level – 10, Duty Cycle – 5, PIP – 140, Cycles/Burst – 200, Time – 4 minutes. Sheared chromatin was immunoprecipitated overnight with the following antibodies: H3K4me3 (Diagenode – C1540003-50), H3K27ac (Abcam – ab4729), H3K4me1 (Diagenode – C15410194), and CTCF (Abcam – ab70303). Antibody chromatin complexes were pulled down with Protein A magnetic beads and washed once in IP wash buffer I. (1% Triton, 0.1% SDS, 150 mM NaCl, 1 mM EDTA, 20 mM Tris, pH 8.0, and 0.1% NaDOC), twice in IP wash buffer II. (1% Triton, 0.1% SDS, 500 mM NaCl, 1 mM EDTA, 20 mM Tris, pH 8.0, and 0.1% NaDOC), once in IP wash buffer III. (0.25 M LiCl, 0.5% NP-40, 1mM EDTA, 20 mM Tris, pH 8.0, 0.5% NaDOC), and once in TE buffer (10 mM EDTA and 200 mM Tris, pH 8.0). DNA was eluted from the beads by vigorous shaking for 20 minutes in elution buffer (100mM $NaHCO_3$, 1% SDS). DNA was de-crosslinked overnight at 65C and purified with MinElute PCR purification kit (Qiagen). DNA was quantified by Qubit and 10 ng DNA was used for sequencing library construction with the Ovation Ultralow Library System V2 (Tecan) using 12 PCR cycles according to the manufacturer's recommendations. Libraries were sequenced with Illumina Nextseq 550, using a paired-end 75bp read configuration.

ATAC-seq

ATAC-seq was performed by using 10^5 bone marrow-derived macrophages (BMDMs) from each condition. Nuclei were isolated with ATAC Lysis Buffer (10mM Tris-HCl pH7.4, 10mM NaCl, 3mM $MgCl_2$, 0.1% IGEPAL). Nuclei from BMDMs were subjected to tagmentation using a Nextera DNA Library Preparation Kit (Illumina). After tagmentation, DNA was purified with a MinElute PCR Purification Kit (Qiagen). Tagmented DNA was then amplified with Phusion high-fidelity PCR master mix (NEB) using 14 PCR cycles. Amplified libraries were purified again with a MinElute PCR Purification Kit. Fragment distribution of libraries was assessed with Agilent Bioanalyzer, and libraries were sequenced on a Novaseq 6000 platform with 150bp paired-end sequencing.

Hi-C

Hi-C was performed by following the reported HiChIP protocol and omitting the protein immunoprecipitation step (3). Briefly, 10^6 bone marrow-derived macrophages were crosslinked with 1% formaldehyde for 10-minutes. Formaldehyde was quenched by the addition of glycine. Nuclei were isolated in lysis buffer (1% Triton x-100, 0.1% SDS, 150 mM NaCl, 1mM EDTA, and 20 mM Tris, pH 8.0) by rotating the cells for 1-hour at 4C. The nuclear pellet was resuspended in 0.5% SDS solution and incubated at 62C for 10-minutes. SDS was quenched by the addition of 10% Triton-X. Chromatin was digested with 8U of

MboI enzyme overnight at 37C. Biotin fill-in was performed for 1-hour at 37C, followed by proximity ligation for 6-hours at room temperature. After proximity ligation, nuclei were subjected to Proteinase K digestion and de-crosslinking overnight at 68C. DNA was column purified (Minelute, Qiagen) and sonicated to a size distribution of 200-500bp. DNA was subjected to streptavidin bead binding to pull-down biotinylated DNA that represents ligation junctions. Tn5 was used to create libraries by on bead tagmentation with sequencing adapters, followed by PCR. Libraries were quantified by Qubit, and size distribution was assessed with Bioanalyzer. Hi-C libraries were sequenced on NovaSeq 6000 with paired-end 150bp configuration. Across the six libraries (two biological replicates per condition), we obtained 243.5–312.4 M total read pairs per sample, yielding 126.6–155.1 M valid unique paired-end reads per sample (average: 141.1 M valid unique pairs). Raw sequencing data are available under GEO accession GSE247946.

Micro Capture-C

Micro Capture-C (MCC) was performed as previously described with the following differences (4). Macrophages were scraped and crosslinked in the presence of 1% formaldehyde (in PBS) for 10-minutes in suspension followed by the addition of 1.5ml Glycine (1M) and further incubated for 5-minutes at room temperature. Cells were washed in ice-cold PBS, counted, and $3x10^6$ aliquots were prepared for nuclei isolation with the following MCC lysis buffer: 10mM Tris-HCl pH 7.5, 10mM NaCl, 3mM $MgCl_2$, 0.1% Tween-20, 0.1% Nonidet P40 Substitute, 0.01% Digitonin, and 1% BSA in nuclease-free H_2O. Each cell aliquot of $3x10^6$ was resuspended in 500ul MCC lysis buffer and incubated on ice for 5-minutes. Cells were spun down, washed in ice-cold PBS, and washed in nuclease-free H2O before proceeding to MNase digestion with 1-2U of MNase for $3x10^6$ cells that went on for 1-hour at 37C while shaking in a thermomixer (Eppendorf) at 550rpm. MNase was stopped by the addition of EGTA. At this point, nuclei were split into two tubes, and end-repair and proximity ligation were performed overnight. On the next day, nuclei were spun and subjected to proteinase K digestion at 65C overnight. DNA was column purified (Minelute, Qiagen) and eluted in 130ul H2O for sonication with Covaris E220 model using the following settings: Fill Level – 10, Duty Cycle – 5, PIP – 140, Cycles/Bursts – 200; sonicated for 4-minutes. DNA was cleaned up with Minelute, quantified and subjected to library preparation with Ovation Ultralow V2 Library Systems (Tecan) using 100ng DNA and 8 PCR cycles. Amplified libraries were subjected to hybridization with biotinylated oligonucleotide pools that were designed by the HyperDesign Team (Roche) and synthesized by Roche. All hybridization reagents were purchased from Roche as part of the KAPA Hyper Prep kit, and hybridization, pull-down, and washes were performed per manufacturer's instructions. Bead-bound libraries were amplified with the PCR reagents from the Ovation Ultralow Library V2 Kit (Tecan). Libraries were quantified and sequenced on a Novaseq 6000 with 2x150bp configuration.

Micro Capture-C analysis

Micro Capture-C analysis was performed as described previously (4). Briefly, adaptor sequences were removed using Trim Galore. Since the majority of the

fragments were sonicated to 200bp, 2x150bp sequencing was performed, and the chimeric fragments were reconstructed into single sequences using FLASH. These reads were then mapped to a specific DNA sequence around the oligonucleotide with BLAT aligner, using an 800-bp region centered on the oligonucleotide as a reference. A second script, MCCsplitter.pl, was employed to split the reads based on their BLAT mapping. Reads that didn't align with any capture oligonucleotide sites were discarded. The segmented reads were subsequently mapped to the genome using Bowtie2. Another script, MCCanalyser.pl, was used to further analyze the data, which is to remove PCR duplicates and to filter out regions with mapping issues. Ligation junctions were identified, and JASPAR was utilized to detect transcription-factor-binding sites at protected DNA regions that exhibited less frequent ligation points. Genome browser track signals in figures represent an average derived from at least six distinct replicates with different MNase concentrations.

RNA-seq analysis

The sequencing data was analyzed using the nf-core RNA-seq pipeline (version 3.9) with default parameters (https://nf-co.re/rnaseq). Initially, FastQC was used for quality control of the fastq files, followed by read trimming via Trim Galore. These processed fastq files were then aligned to the mm10 mouse genome with STAR aligner. Subsequently, Salmon generated a gene-by-sample count matrix for further analysis. After performing PCA on variance-stabilized read counts, batch effects from animals were addressed using the removeBatchEffect function from the limma package (5). Differential gene expression analysis was performed using DESeq2 (6) with a multi-factor design (~ batch + condition) that accounts for batch effects across biological replicates. For the IL-4 priming analysis and signal integration analyses, pairwise comparisons were made between each treatment condition and the untreated control. Significance thresholds were $|\log_2 FC| \geq 0.5$ and Benjamini-Hochberg FDR < 0.05. For visualization, a heatmap was constructed by aggregating differential genes of different treatment conditions against control groups and standardizing expressions. Genes were clustered using k-means clustering, considering Pearson correlation between rows. QIAGEN Ingenuity Pathway Analysis and clusterProfiler (version 4.8.3) were used for pathway analysis of genes in each cluster.

Upstream regulator analysis

Upstream regulator prediction was performed using QIAGEN Ingenuity Pathway Analysis (IPA, QIAGEN Inc.). Gene lists from each cluster were uploaded to IPA, and the Upstream Regulator Analysis tool was used to identify potential upstream regulators (transcription factors, cytokines, and chemical compounds) that could explain the observed gene expression changes. Regulators were ranked by overlap p-value (Fisher's exact test) and activation z-score. The top 3 cytokine and chemical drug regulators are shown in Figure 1F. Regulators with adjusted p-value < 0.01 were considered significant.

ChIP-seq analysis

Sequencing adapters were trimmed using Trimmomatic and the reads were aligned to the mm10 reference genome using Bowtie2 with the --very-sensitive option (7). The BAM files obtained were sorted based on their genomic coordinates using Samtools to eliminate PCR duplicates. After alignment and sorting, PCR duplicates were identified and removed using Picard. The BAM files were then converted to BED files using bamToBed and intersected with the mm10 blacklist. To normalize the output bedgraph files, genomeCoverageBed from the Bedtools package was used, and the resulting files were converted to bigwig format using bedGraphToBigWig from the UCSC tools. Peaks were identified from the BED file using MACS2 (8) with a false discovery threshold of 0.05 (-q 0.05). To create a non-overlapping consensus peak set, overlapping peaks with lower significance across all samples were iteratively eliminated. By counting the overlaps of reads in each sample with the consensus peak set, a peak-by-sample matrix was generated. Differential peak analysis was then performed on the peak-by-sample count matrix, applying an absolute log2 fold change threshold of 0.5 and a p-value threshold of 0.05.

Hi-C analysis

We utilized the nf-core/hic (2.0.0) pipeline (9) for Chromosome Conformation Capture (Hi-C) data analysis, built on the Nextflow framework with Docker/Singularity containers ensuring reproducibility. Initial read quality was assessed using FastQC. HiC-Pro was used for the core processing, including mapping with bowtie2, interaction product detection, and contact map generation (10). A final quality report was generated with MultiQC. Genome-wide contact maps were created and normalized with JuiceBox (11). We used HICCUPS (12) from the Juicer pipeline to call chromatin loops at 25 kb resolutions. Loops were identified using standard parameters, with a significance threshold of FDR < 0.10. Knight-Ruiz (KR) normalization was applied to all contact matrices before loop calling. The loops with 25k resolution from all conditions were initially combined into a union set and HiC matrix raw counts were extracted from .hic files with hictoolsr (v 1.1.2). Differential loop heatmap was constructed by testing each condition against the control group using DESeq2 with the loop-counts matrix constructed in the previous step (abs($\log_2$ fold change) > 0.5 and p-value < 0.1). The aggregate signal of the HiC matrices were generated with Coolpup.py (13). Briefly, the .hic contact matrices were converted into .cool format with cooltools (14), and the average signal of each differential loop group was calculated with pileup() function with default parameters.

Loop anchor gene enrichment permutation test

Gene TSS were extended by 100 kb on both sides, and we tested whether they overlapped with any of the loop anchors to be considered regulated by the chromatin loops. Permutation tests were performed with the permTest function in the regioneR package (15). Specifically, we calculated the number of overlaps between the target gene with extended TSS and loop anchors and then generated 5,000 or 1,000 random background genes with extended TSS regions using the

randomizeRegions function to establish the background distribution and perform the statistical tests.

Integrative analysis of RNA-, ATAC-, and ChIP-seq

Chromatin accessibility and ChIP binding signal histograms and heatmaps around the TSS of differentially expressed genes were generated using featureAlignedDistribution from ChIPseeker R package (16). A 3kb window was used to aggregate ATAC and ChIP track signals at differentially accessible sites around the upstream and downstream 100kb of the TSS.

Statistical Analyses

Statistical analyses were performed in R or GraphPad Prism. qPCR measurements were presented as means +/- SD and three biological replicates were performed. The exact replicate numbers are indicated in the figure legends for each experiment. On the bar graphs, significant changes were determined by two-tailed, unpaired t-test at $p<0.05$. Differential loops from Hi-C were called by $p<0.1$, Log_2 $FC\geq0.5$. Differential chromatin accessibility and ChIP-seq analyses were performed with the following parameters: $p<0.05$, Log_2 $FC>0.5$, unless specified otherwise. Differential gene expression analyses were performed with the following parameters: $FDR<0.05$, $Log_2FC>0.5$. Statistical parameters are reported in the figure legends and in the results section.

Figures

Figure S1.

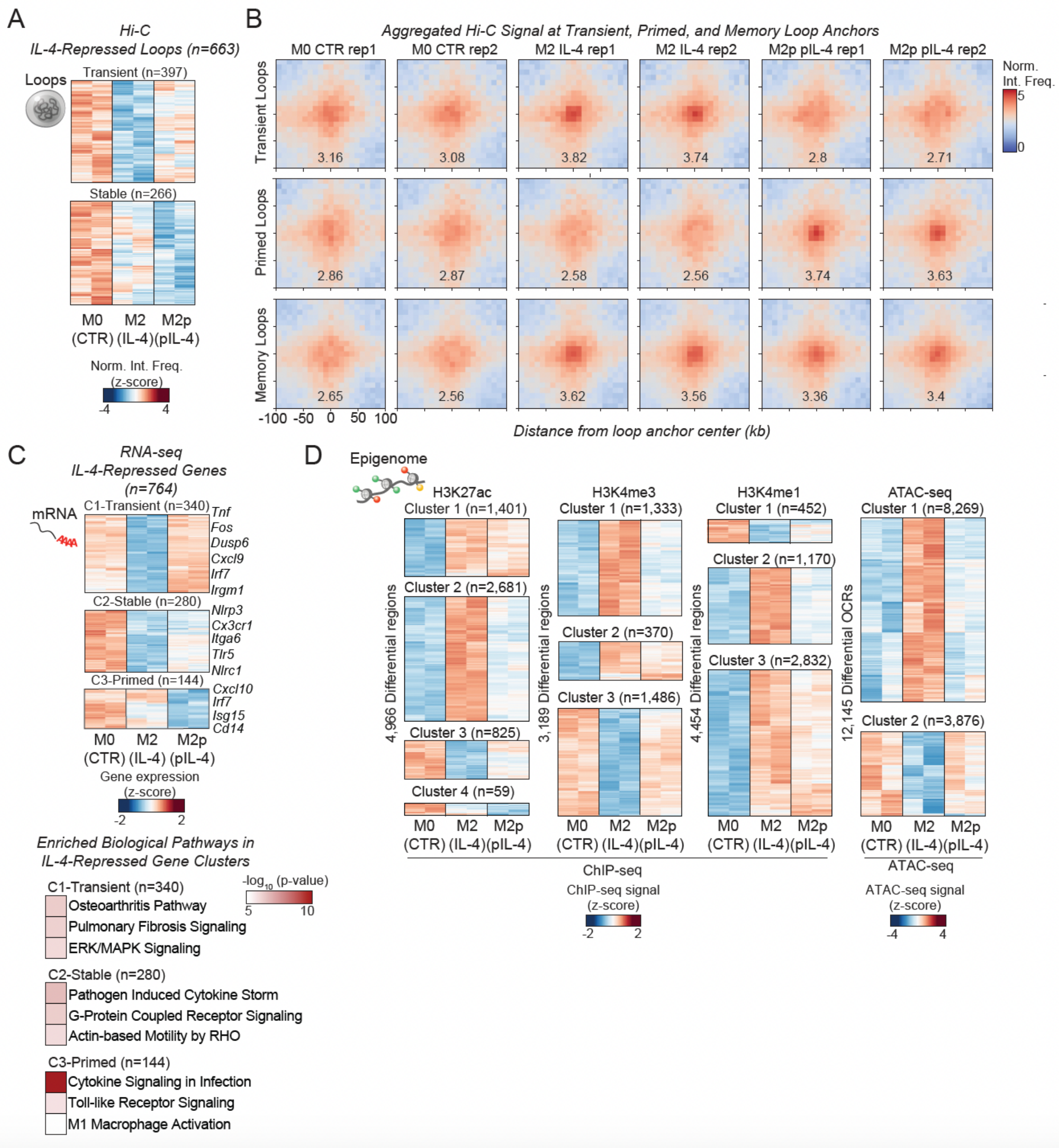

Figure S1. Characterization of transcriptional and 3D structural changes during macrophage priming by IL-4. Related to Figure 1. (A) Heatmap representation of IL-4-repressed chromatin loops determined by Hi-C. **(B)** Aggregate normalized Hi-C signal at IL-4-induced chromatin loop anchors, highlighting interaction patterns across the indicated groups. **(C)** Heatmap representation of IL-4-repressed mRNA transcript groups (top). Small heatmaps represent the results of Ingenuity pathway analysis (details in Methods) for each RNA-seq gene cluster, and the top 3 biological terms are shown (bottom). **(D)** Heatmap representation of genomic regions with differential histone enrichment profiles and ATAC-seq profiles in the indicated conditions (IL-4 mediated priming model).

Figure S2.

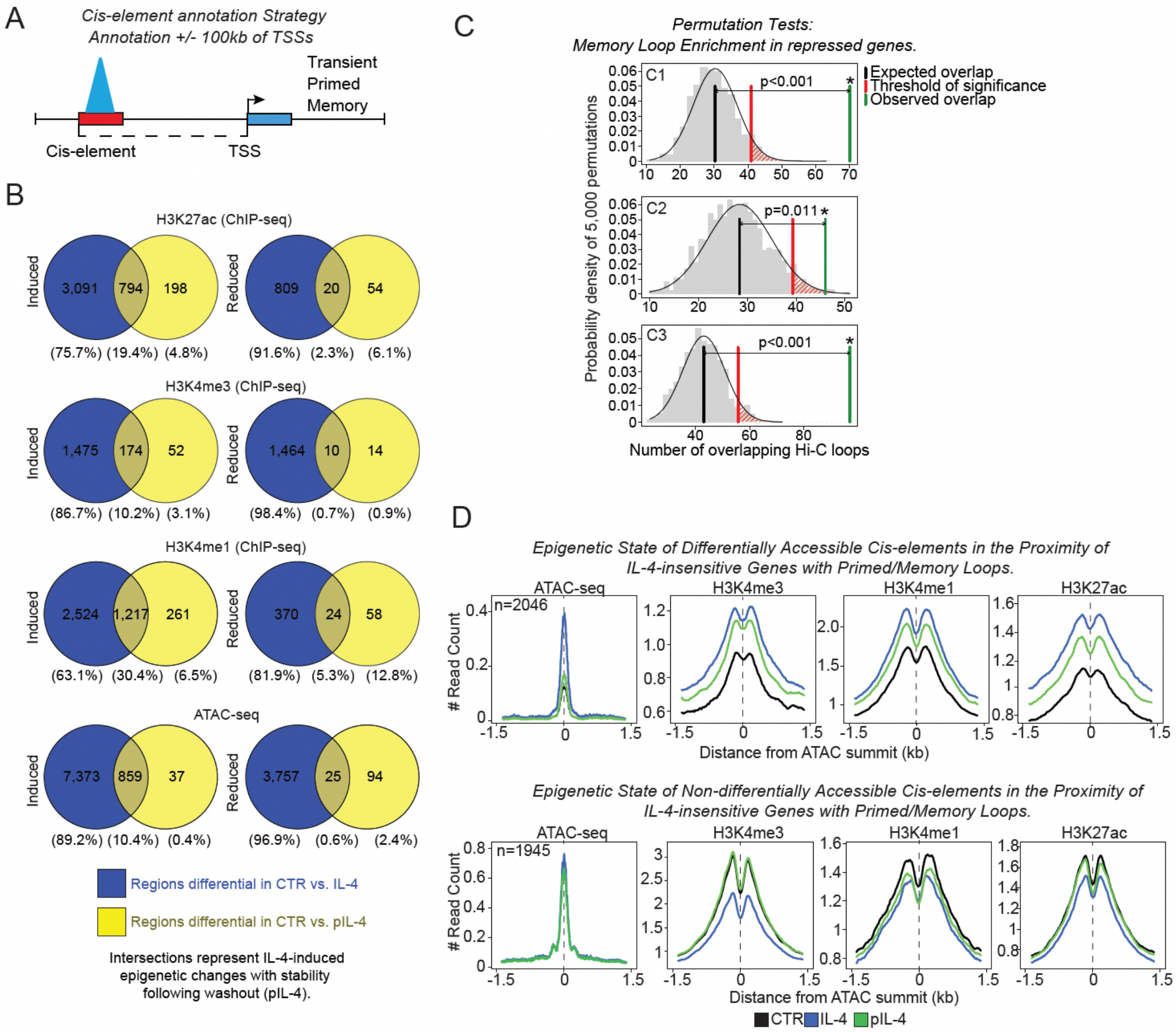

Figure S2. Epigenetic characterization of macrophage priming by IL-4. Related to Figure 1. (A) Scheme of annotation of distant IL-4-induced open chromatin regions to differentially expressed genes in a +/-100kb genomic window around their transcription start sites. **(B)** Venn diagrams depict the IL-4-induced and -reduced histone modification landscape and identify the stable effects of IL-4 that are related to memory formation. Percentage wise distribution of the genomic regions across the CTR vs. IL-4 and CTR vs. pIL-4, and their overlap is plotted. **(C)** Permutation tests assessing the enrichment of Memory loops in the proximity of IL-4-repressed gene groups. Red vertical line: significance threshold (α = 0.05). Black vertical line: expected overlap based on random background. Green vertical line: observed overlap. **(D)** Epigenetic profiles of *cis*-regulatory elements associated with IL-4–insensitive genes linked to Primed and Memory loops. Top panels show differentially accessible regions, whereas bottom panels show non-differentially accessible regions. Aggregate ATAC-seq, H3K4me3, H3K4me1, and H3K27ac signal centered on ATAC summits are shown for CTR, IL-4, and pIL-4 conditions.

Figure S3.

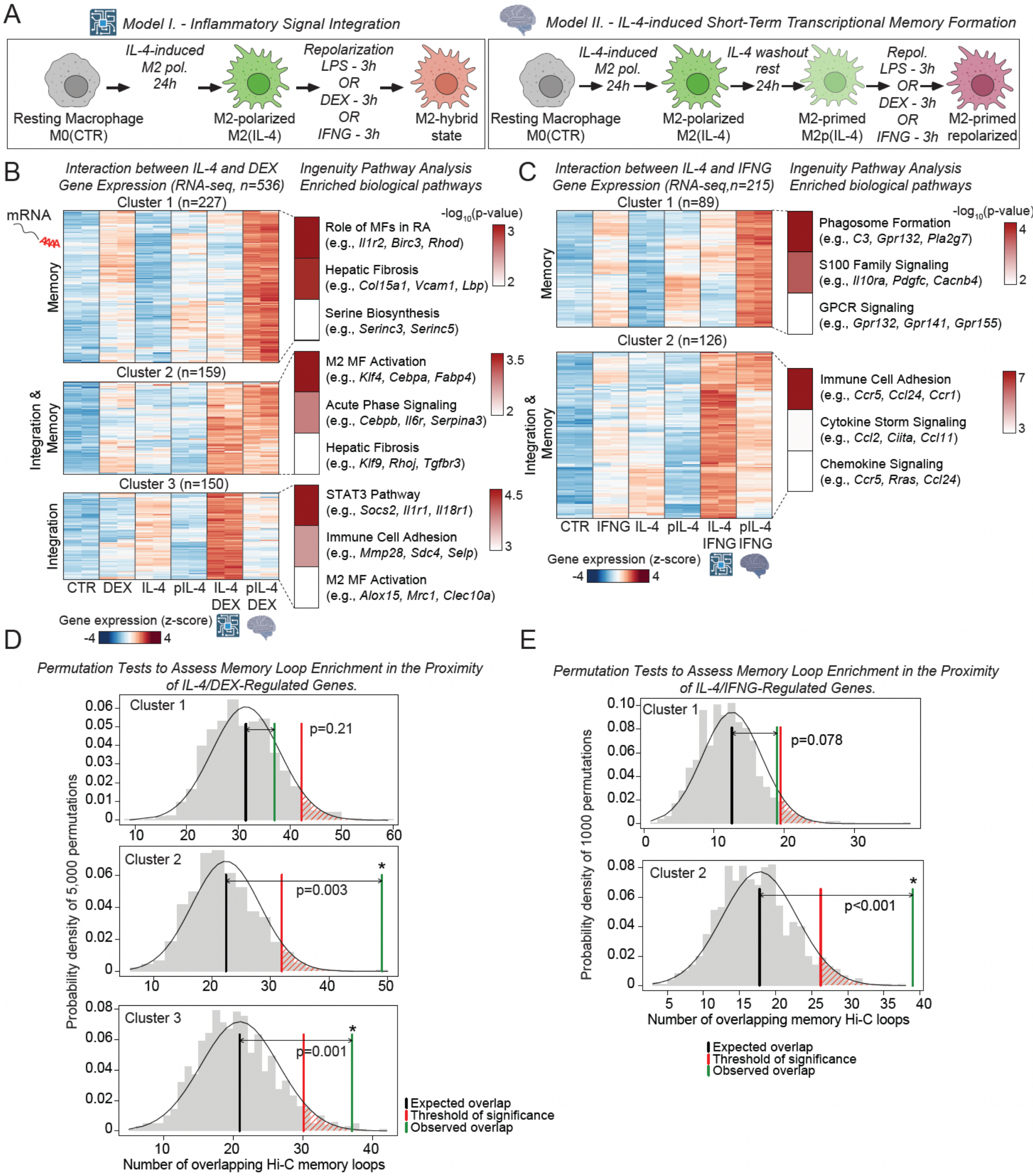

Figure S3. IL-4 priming enhances DEX and IFNG-induced gene programs. Related to Figure 3. (A) Schematics of the two model systems used to study Inflammatory signal integration (Model I.) and Short-term Memory formation (Model II.). Heatmap representation of differentially expressed genes (DEGs) detected by RNA-seq for **(B)** DEX and **(C)** IFNG treatment. Small heatmaps represent the results of Ingenuity pathway analysis for each RNA-seq gene cluster, and the top 3 biological terms are shown (middle). Permutation test results that assess the enrichment of Memory loops in the different groups of DEGs in the **(D)** IL-4 - DEX, and **(E)** IL-4 - IFNG treatment paradigm. Red vertical line: significance threshold (α = 0.05). Black vertical line: expected overlap based on random background. Green vertical line: observed overlap.

Figure S4.

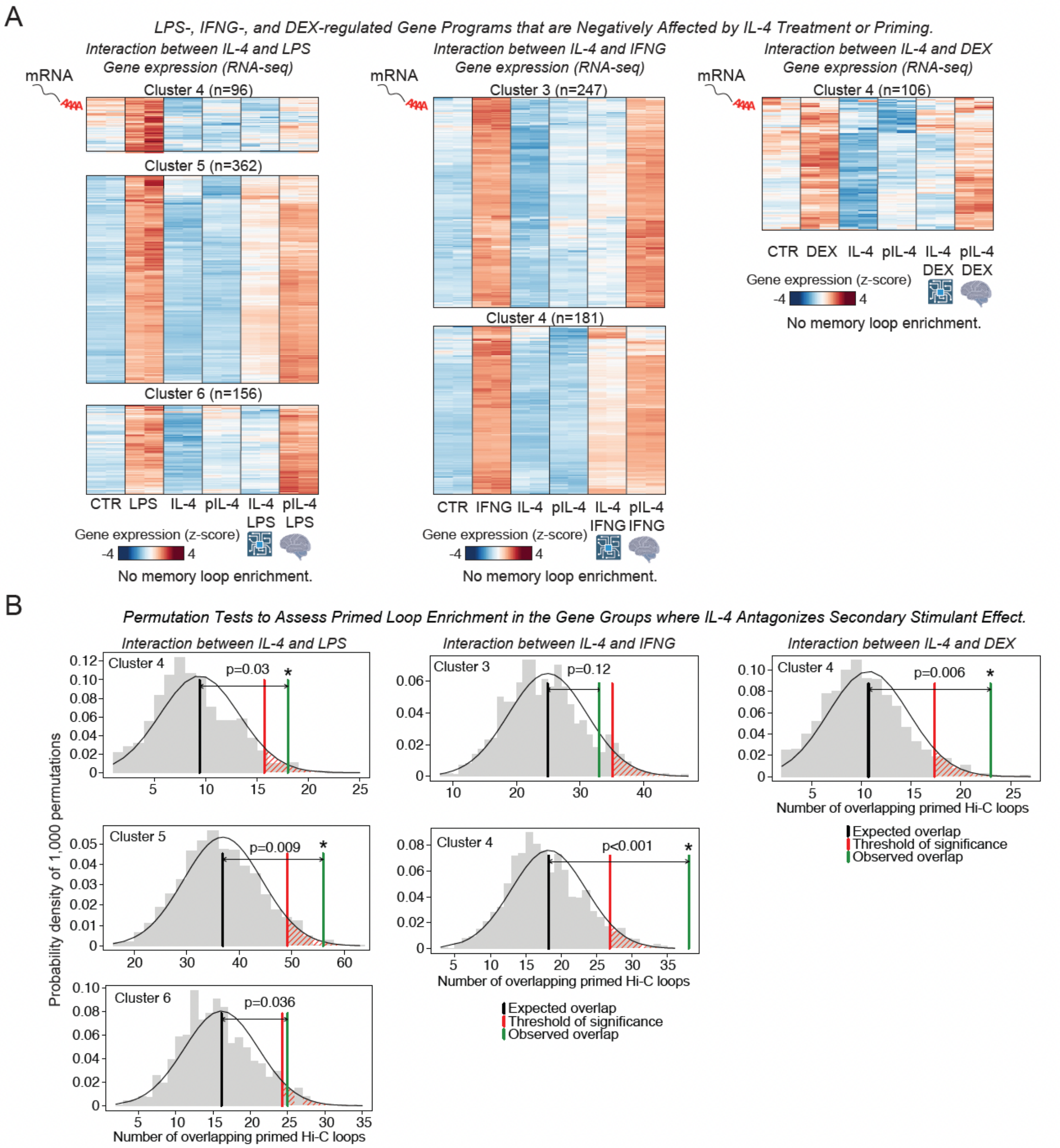

Figure S4. IL-4 priming suppresses a subset of secondary signal-responsive transcriptional programs. Related to Figure 3. (A) Heatmap representation of differentially expressed genes (negatively affected by IL-4) in the indicated treatment paradigms and models. **(B)** Permutation tests of each gene group in Fig S4A. with primed loops. Red vertical line: significance threshold (α = 0.05). Black vertical line: expected overlap based on random background. Green vertical line: observed overlap.

Figure S5.

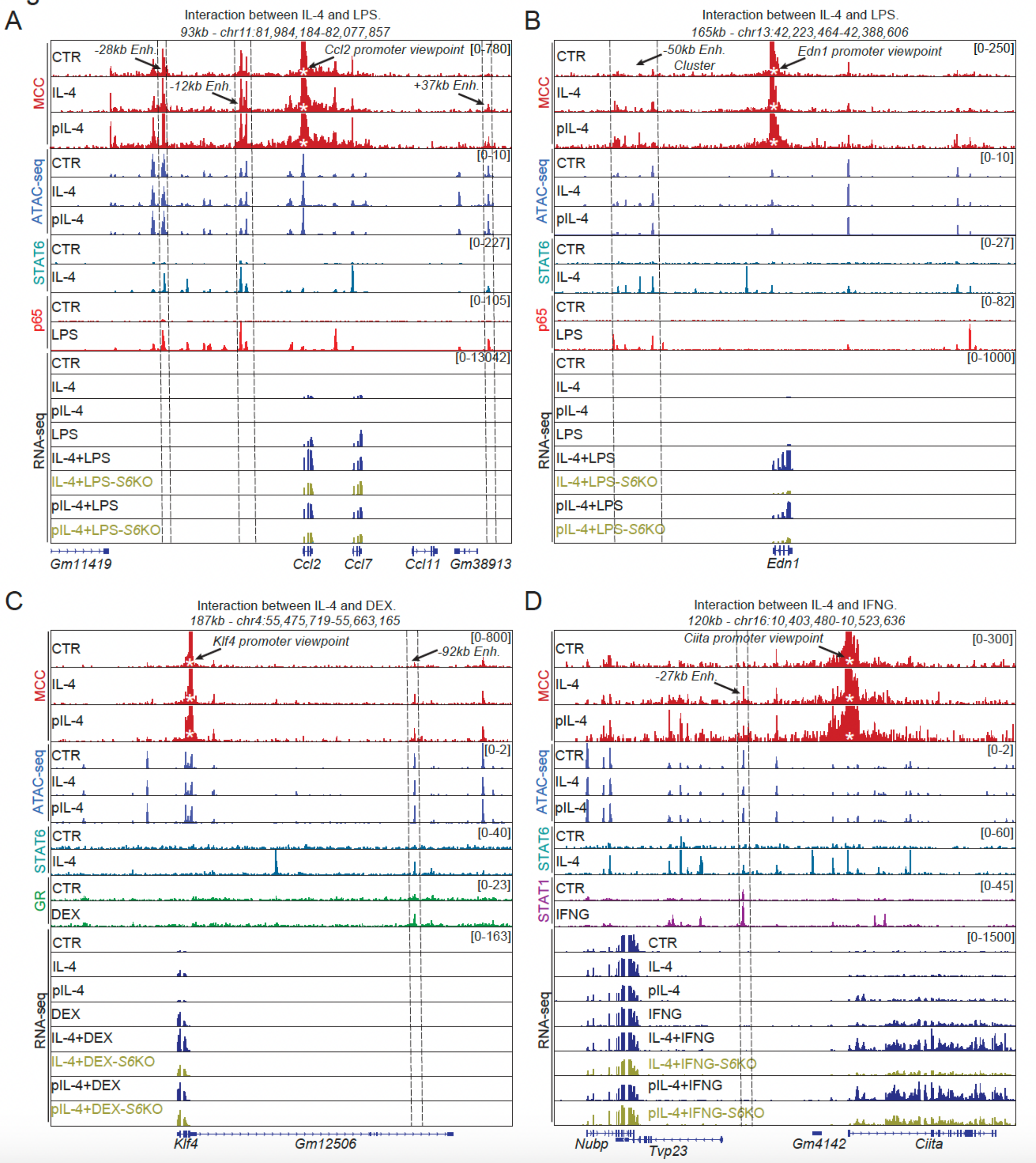

Figure S5. **IL-4 priming connects secondary stimulant-responsive enhancers to their cognate gene promoters. Related to Figures 3 and S3.** Genome browser tracks showing genomic datasets in the **(A)** *Ccl2*, **(B)** *Edn1* in response to LPS, **(C)** *Klf4* in response to DEX, and **(D)** *Ciita* in response to IFNG.

Figure S6.

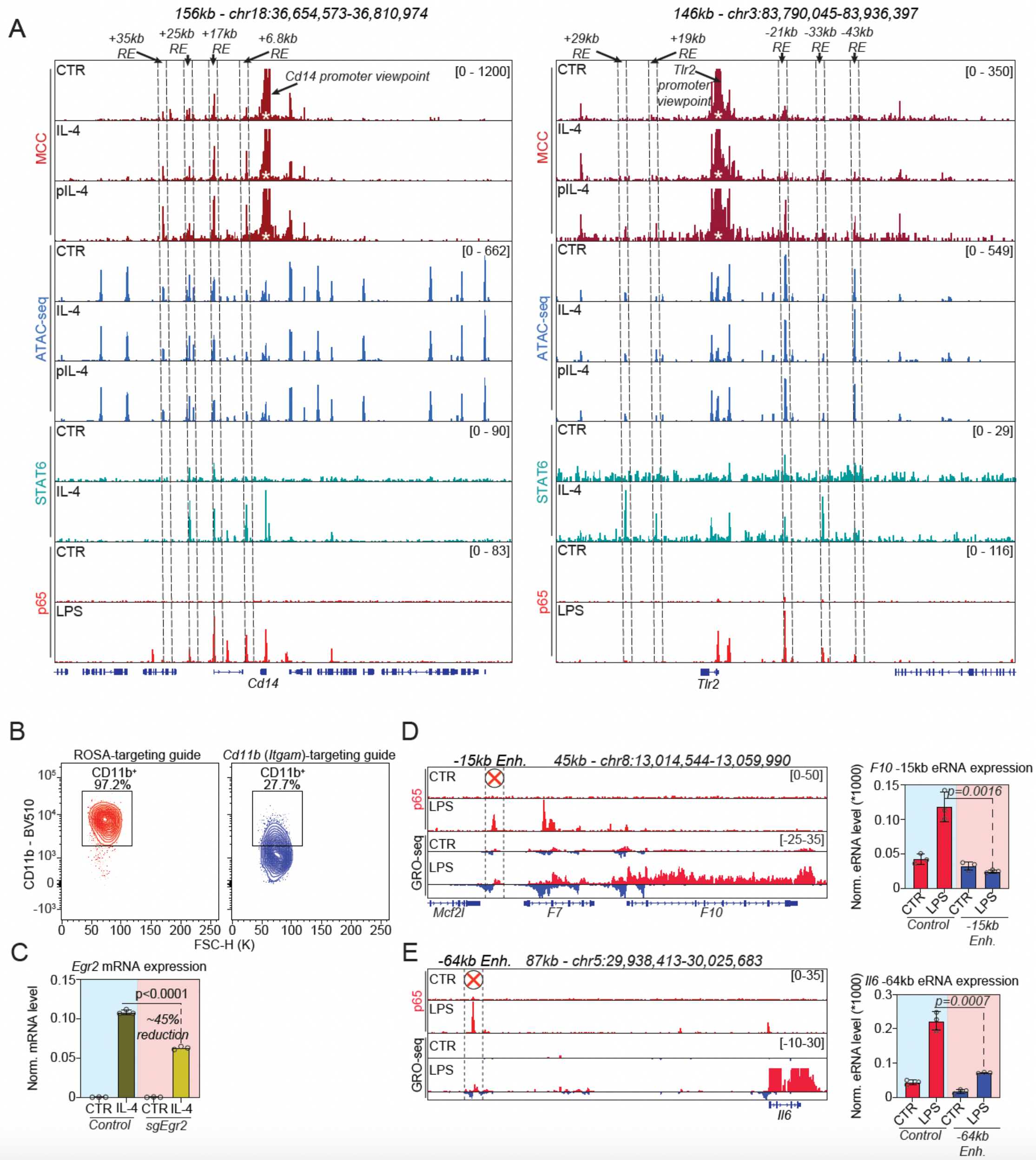

Figure S6. Transcriptional memory formation and signal integration is linked to specific memory loop anchor points. Related to Figures S3, S4, and S5. (A) Genome browser views of the indicated datasets in the *Cd14* and *Tlr2* genomic loci. **(B)** FACS data of CD11b expression on the cell surface of macrophages in the presence of a control guide RNA (ROSA-targeting) and Cd11b-targeting guide during a CRISPR optimization experiment. **(C)** RT-qPCR measurements of *Egr2* mRNA expression after control (ROSA-targeting) and *Egr2*-targeting guide and Cas9 electroporation. Significant differences were determined by two-tailed, unpaired t-test at $p<0.05$. **(D)** Genome browser snapshot of p65 ChIP-seq and GRO-seq datasets that guided the design of enhancer RNA-specific primers to detect the effects of guide RNAs in CRISPR experiments that targeted these *cis*-elements. RT-qPCR measurements of *F10 -15kb* eRNA expression after control (ROSA-targeting) and *cis*-element-targeting guide and Cas9 electroporation. **(E)** Same as panel D, but for the *Il6 -64kb cis*-element. Significant differences were determined by two-tailed, unpaired t-test at $p<0.05$.

SI Datasets

Dataset S1 – Differentially expressed genes in Figure 1C.
Dataset S2 – Differentially expressed genes in Supplemental Figure 1C.
Dataset S3 – Gene groups reported in the Venn diagram in Figure 1F.
Dataset S4 – Differentially expressed genes in Figure 3B.
Dataset S5 – Differentially expressed genes in Supplemental Figure 3B.
Dataset S6 – Differentially expressed genes in Supplemental Figure 3C.
Dataset S7 – Differentially expressed genes in Supplemental Figure 4A.
Dataset S8 – Primer and guide RNA sequences used in the study.

SI References

1. E. C. Freund, et al., Efficient gene knockout in primary human and murine myeloid cells by non-viral delivery of CRISPR-Cas9. J. Exp. Med. 217 (2020).

2. B. Daniel, B. L. Balint, Z. S. Nagy, L. Nagy, Mapping the genomic binding sites of the activated retinoid X receptor in murine bone marrow-derived macrophages using chromatin immunoprecipitation sequencing. Methods Mol. Biol. 1204, 15–24 (2014).

3. M. R. Mumbach, et al., HiChIP: efficient and sensitive analysis of protein-directed genome architecture. Nat. Methods 13, 919–922 (2016).

4. P. Hua, *et al.*, Defining genome architecture at base-pair resolution. *Nature* 595, 125–129 (2021).

5. M. E. Ritchie, et al., limma powers differential expression analyses for RNA-sequencing and microarray studies. Nucleic Acids Res. 43, e47 (2015).

6. M. I. Love, W. Huber, S. Anders, Moderated estimation of fold change and dispersion for RNA-seq data with DESeq2. Genome Biol. 15, 550 (2014).

7. B. Langmead, S. L. Salzberg, Fast gapped-read alignment with Bowtie 2. Nat. Methods 9, 357–359 (2012).

8. Y. Zhang, et al., Model-based analysis of ChIP-Seq (MACS). Genome Biol. 9, R137 (2008).

9. P. A. Ewels, et al., The nf-core framework for community-curated bioinformatics pipelines. Nat. Biotechnol. 38, 276–278 (2020).

10. N. Servant, et al., HiC-Pro: an optimized and flexible pipeline for Hi-C data processing. Genome Biol. 16, 259 (2015).

11. N. C. Durand, et al., Juicebox provides a visualization system for Hi-C contact maps with unlimited zoom. Cell Syst. 3, 99–101 (2016).

12. S. S. P. Rao, et al., A 3D map of the human genome at kilobase resolution reveals principles of chromatin looping. Cell 159, 1665–1680 (2014).

13. I. M. Flyamer, R. S. Illingworth, W. A. Bickmore, Coolpup.py: versatile pile-up analysis of Hi-C data. Bioinformatics 36, 2980–2985 (2020).

14. Open2C, et al., Cooltools: Enabling high-resolution Hi-C analysis in Python. PLoS Comput. Biol. 20, e1012067 (2024).

15. B. Gel, et al., regioneR: an R/Bioconductor package for the association analysis of genomic regions based on permutation tests. Bioinformatics 32, 289–291 (2016).

16. G. Yu, L.-G. Wang, Q.-Y. He, ChIPseeker: an R/Bioconductor package for ChIP peak annotation, comparison and visualization. Bioinformatics 31, 2382–2383 (2015).
